# Adhesion, forces and the stability of interfaces

**DOI:** 10.3762/bjoc.15.12

**Published:** 2019-01-11

**Authors:** Robin Guttmann, Johannes Hoja, Christoph Lechner, Reinhard J Maurer, Alexander F Sax

**Affiliations:** 1 Department of Chemistry, University of Graz, Heinrichstrasse 28, 8010 Graz, Austria; 2 Present address: Physics and Materials Science Research Unit, University of Luxembourg, 1511 Luxembourg, Luxembourg; 3 Department of Chemistry and Centre for Scientific Computing, University of Warwick, Gibbet Hill Road, Coventry, CV4 7AL, United Kingdom

**Keywords:** adhesion energy, adhesive force, dispersion interaction, weak molecular interaction

## Abstract

Weak molecular interactions (WMI) are responsible for processes such as physisorption; they are essential for the structure and stability of interfaces, and for bulk properties of liquids and molecular crystals. The dispersion interaction is one of the four basic interactions types – electrostatics, induction, dispersion and exchange repulsion – of which all WMIs are composed. The fact that each class of basic interactions covers a wide range explains the large variety of WMIs. To some of them, special names are assigned, such as hydrogen bonding or hydrophobic interactions. In chemistry, these WMIs are frequently used as if they were basic interaction types. For a long time, dispersion was largely ignored in chemistry, attractive intermolecular interactions were nearly exclusively attributed to electrostatic interactions. We discuss the importance of dispersion interactions for the stabilization in systems that are traditionally explained in terms of the “special interactions” mentioned above. System stabilization can be explained by using interaction energies, or by attractive forces between the interacting subsystems; in the case of stabilizing WMIs, one frequently speaks of adhesion energies and adhesive forces. We show that the description of system stability using maximum adhesive forces and the description using adhesion energies are not equivalent. The systems discussed are polyaromatic molecules adsorbed to graphene and carbon nanotubes; dimers of alcohols and amines; cellulose crystals; and alcohols adsorbed onto cellulose surfaces.

## Introduction

Any change of the state of motion of a particle, described in an inertial frame, is caused by a force acting on the particle. The change of motion, i.e., the acceleration, causes a change of the position of the particle in space. If in a system of particles all particles exert forces on each other, these forces are called internal forces. In the simplest system, particle A at position **r**_A_ exerts a force **F**_A→B_ on particle B and particle B at position **r**_B_ exerts a force **F**_B→A_ on particle A. Both forces obey Newton’s third law, **F**_A→B_ = −**F**_B→A_, expressed by the Latin phrase “actio est reactio”. This process of mutually exerting internal forces is called an interaction. If the internal forces are conservative, a potential function *V*_AB_(**r**_A_,**r**_B_) can be defined, and both internal forces can be calculated as gradients with respect to the position of the particles. With the advent of the science of energetics, as promoted by William Rankine, forces were nearly completely replaced by potentials in the description of interacting systems, the term “force” remained as a synonym for interaction. When we speak of a force in this paper, however, we always mean the physical vector quantity or its magnitude.

The obvious advantage of using the scalar quantity, energy, instead of the vector quantity, force, is that it is simpler to describe and categorize system stabilization by using properly defined stabilization energies calculated as differences in the values of the energy functions. In chemistry, stabilizing interactions are roughly classified as strong or weak according to the magnitude of stabilization energies [1]. Strong interactions are 1) Coulomb interactions in ionic solids ranging between 600 kJ/mol (CsI) and 3900 kJ/mol (MgO), 2) covalent interactions in molecules ranging between about 150 kJ/mol (I_2_) and 950 kJ/mol (N_2_), and 3) metallic interactions ranging between 65 kJ/mol (Hg) and 850 kJ/mol (W). Ionic and metallic interactions are the interactions in extended systems, mostly solids, whereas covalent interactions are between molecular subsystems (fragments, radicals) at localized positions, mostly atom positions. Interactions between atoms or small molecules with closed-shell electron configurations having stabilization energies of up to 50 kJ/mol are typical weak interactions. They are smaller by a factor of roughly ten than Coulomb interactions or covalent bonding.

It is a characteristic of attractive, weak molecular interactions (WMIs) that the molecules involved retain their integrity. This may mean one of three things: 1) that the geometries of the interacting molecules differ very little from the equilibrium geometries of the isolated species, e.g., an interacting molecule changes only its conformation; 2) that the neutral molecules do not undergo an electron-transfer interaction to form cation–anion pairs; or 3) that there is no significant change in the electronic structure of the interacting molecules, such as that caused by electronic excitation or covalent bonding. The absence of covalent bonding (case 3) is also the reason for using the term “non-covalent interaction”, another frequently used term is “weak intermolecular interaction”. Both terms have disadvantages. Weak intermolecular interaction does not cover those cases in which intramolecular interactions cause stabilization, e.g., when a large *n*-alkane changes from the linear to the hairpin structure; non-covalent interaction, on the other hand, does not exclude creation and stabilization of cation–anion pairs or zwitterions and their stabilization by Coulomb interaction. Weak molecular interaction is certainly the best term for describing any attractive interaction in which the interacting subsystems retain their integrity.

WMI does not have a single physical cause. Instead, several basic interactions are responsible for the interactions between molecules, which can be seen as extended charge distributions consisting of nuclei and electrons. When interactions between saturated molecules in their electronic ground states are considered, there are four basic interactions: 1) electrostatics, which are the interactions between static multipoles without any charge shift in the interacting molecules; 2) induction or polarization interactions, which are those between static multipoles in one and multipoles in the other molecule that are induced by charge shifts; 3) dispersion interactions, which are those between non-static multipoles in one molecule and induced multipoles in the other molecule; and 4) exchange repulsions or Pauli repulsions, which describe the tendency of electrons to avoid coming spatially close due to their Fermion character, not due to their charge [2]. Electrostatics and induction can be explained with classical physics, whereas dispersion and exchange repulsions are pure quantum effects. Induction and dispersion are also called polarization interactions, because both involve polarizations in at least one interacting molecule. An intricate aspect of WMI is that the four basic interactions may contribute with different weights; moreover, in each group, different “flavors” can be found due to the different distance dependencies of the various multipole–multipole interactions. The WMI for a certain pair of interacting molecules is like a cocktail composed of four basic ingredients, the characteristics of the cocktail are due not only to the different bar measures of the basic ingredients, but also due to their different flavors.

Hydrogen bonding is a typical WMI. As such, it is composed of the abovementioned basic interactions, each having its own strength and range. Nevertheless, it is common practice in chemistry to speak about hydrogen bonding as if it was indeed a genuine basic interaction rather than a composed interaction. Instead of stressing the different compositions of the basic interactions, chemists speak of strong, moderate or weak hydrogen bonding [3]; sometimes even further divisions are made [4]. Hydrogen bonding was introduced nearly 100 years ago to explain the stabilization of complexes of, e.g., water, alcohol or amine molecules. The stabilization was first explained solely by electrostatic attraction, but this simplistic view was already corrected in 1952 by Coulson [5], who stressed the need to also consider induction and dispersion as attractive interactions. Nevertheless, even today it is more often claimed than actually demonstrated that hydrogen-bonded complexes are predominantly stabilized by electrostatics [6]. If any other interaction but electrostatics is considered, it is “charge-transfer”, which suggests that the dimer stabilization is caused by an electron transfer, although this would mean the creation of a cation–anion pair and, thus, a loss of molecular integrity. What is meant, however, is a polarization of the electron density due to a charge shift, which is covered by the basic induction interactions [2]. Although dispersion interaction is a ubiquitous attractive interaction, it is frequently considered to be less important than electrostatics when explaining hydrogen bonding. However, we have shown that this is not the case in our studies on the stabilization of alcohol and amine dimers [7–8].

Another type of WMI is the hydrophobic interaction, which was introduced by Kauzmann [9] to explain protein folding in analogy with the transfer of a non-polar solute from water into a non-polar solvent. This process was attributed to the poor solubility of the solute in water. Wolfenden and Lewis [10], on the other hand, assumed “that a strong favorable interaction among alkane molecules in liquid alkanes gives a strong favorable transfer energy for passage of an alkane from vapor into liquid alkane”, explaining the poor solubility of hydrocarbons in water and the good solubility of alkane molecules in liquid alkane [11]. Nonetheless, this interaction is nothing more than a dispersion-dominated WMI.

On the other hand, electrostatic interactions are often ignored, unless the interacting molecules have obvious dipolar structures. For example, the fact that there is electrostatic interaction between the quadrupoles of benzene molecules is mostly ignored or not even known. Instead, attraction is attributed to π–π interactions or CH–π interactions of unclear physical origin. That deformation of molecules induces static multipoles is also not well known; the bending of non-polar planar molecules that have a quadrupole as their lowest static multipole (e.g., polyaromatic hydrocarbons) induces a dipole moment; likewise, when a spherical charge distribution is deformed to an ellipsoid, a quadrupole is induced. Discussion of WMI, as found in the chemical literature, often suffers from a profound confusion of tongues due to the preference of a folkloristic [12] instead of a physically sound language.

With respect to extended systems, one has to consider an important modification of the theory of WMI. The standard calculation of the contributions to WMI is based on the multipole expansion of the charge distributions involved with respect to a single expansion center. This is justified for small molecules, but this expansion slowly converges or fails for large molecular systems. In molecular orbital theory, the slow convergence of single-center expansions of molecular orbitals, which is mathematically equivalent to the multipole expansion, was cured by the use of atom-centered basis functions in the linear combination of atomic orbitals (LCAO) approximation. This approximation allows the expansions to be stopped at much smaller angular momentum quantum numbers than in a single-center expansion. In the context of WMI, replacements of single-center expansions by multicenter expansions are termed distributed multipole analysis, distributed polarizabilities, and distributed dispersion interaction [2]. The possibility of calculating electrostatic, induction and dispersion interactions by dividing molecules into subsystems, mostly atoms or atom groups, which are characterized by their own short multipole expansion, together with the short range of attractive induction and dispersion interactions in particular, explains our findings of an approximate additivity of the stabilization energy and the adhesive forces [13–15]. Adhesion is the term for the attractive interaction between unlike subsystems, e.g., a graphene sheet and adsorbed molecules, whereas the attractive interaction between like subsystems, e.g., graphene sheets in graphite, is called cohesion. Nonetheless, the basic interactions are the same for adhesion and cohesion.

We attributed the additivity to the “near-sightedness” of WMIs, and defined the contact zone of two interacting molecules as the set of all atom pairs making non-negligible contributions to the adhesion energy and adhesive forces. We showed that the contact zone is a useful means for discussing the origin of stabilization of parallel alkane chains, as well as the stabilization of aromatic molecules adsorbed to graphene or carbon nanotubes. Furthermore, we found that the stabilization energy of an adsorbent and several small adsorbate molecules increases when the latter are in close contact with each other. This cooperative effect agrees well with the approximate isotropy of dispersion interactions.

In this paper, we discuss the implications of WMIs on structure and stability of different systems we studied in the past. We discuss the physical origin of WMIs, that is, their composition of different basic interaction types laying the focus on the role of dispersion interactions. We show that dispersion interactions are essential for the correct description of the structure and stability of systems composed of subsystems, such as dimers or clusters of small molecules, or interfaces between large adsorbents and adsorbates of different sizes. We discuss the different roles of adhesion energies and adhesive forces and friction forces for the description of the stability of condensed matter systems, and we show that use of the vector quantity force is essential for the understanding of mechanical stability of solids, and for many properties such as boiling point or viscosity of liquids.

## Basics of Weak Molecular Interaction

### Description of interaction through forces and potentials

Interactions in a system consisting of two or more subsystems cause changes of the spatial positions of the subsystems relative to each other. Attractive interactions reduce the distance between the centers-of-mass of two subsystems, whereas repulsive ones increase the distance. There may also be changes in the relative orientation of the subsystems due to rotations without any change in the distance between the centers of mass. The internal forces that each subsystem exerts on the others change their atomic positions; thus changes of the atomic positions are an indicator of interactions in the system.

For the moment, we assume that the two subsystems are structureless and completely described by the center-of-mass coordinates **R**_A_ and **R**_B_, the structure of the total system is presented by **R** = (**R**_A_, **R**_B_). Forces **F**_A→B_, exerted by subsystem A on subsystem B, and **F**_B→A_, exerted by subsystem B on subsystem A, depend in general on both subsystems, **F**_A→B_ = **F**_A→B_(**R**_A_, **R**_B_). Each is the negative of the other, the relation **F**_A→B_(**R**_A_, **R**_B_) = −**F**_B→A_(**R**_A_, **R**_B_), expressing Newton’s third law, can also be written as **F**_A→B_ + **F**_B→A_ = 0. Since this relation defines balanced forces, all internal forces are balanced forces.

An alternative way of describing interaction in a system uses a potential energy function (PEF) *V *^int^(**R**_A_, **R**_B_), called the interaction potential, for structureless subsystems, the potential depends only on the distance *r* = |**R**_B_ − **R**_A_| between the particles, *V *^int^(**R**) = *V *^int^(*r*). The internal forces are the negative gradients of the PEF with respect to the center-of-mass coordinates, **F**_A→B_(**R**_A_, **R**_B_) = 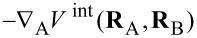 and **F**_B→A_(**R**_A_, **R**_B_) = 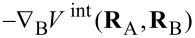. All elementary electrostatic potentials are strictly monotonic functions in *r*, such as reciprocal powers or exponentially decreasing functions, and they obey the asymptotic boundary condition lim*_r_*_→∞_*V*(*r*) = 0. For all finite values of *r*, they have either only positive or only negative values. Monotonically decreasing PEFs represent repulsive interactions, monotonically increasing PEFs represent attractive interactions.

**Figure 1:**
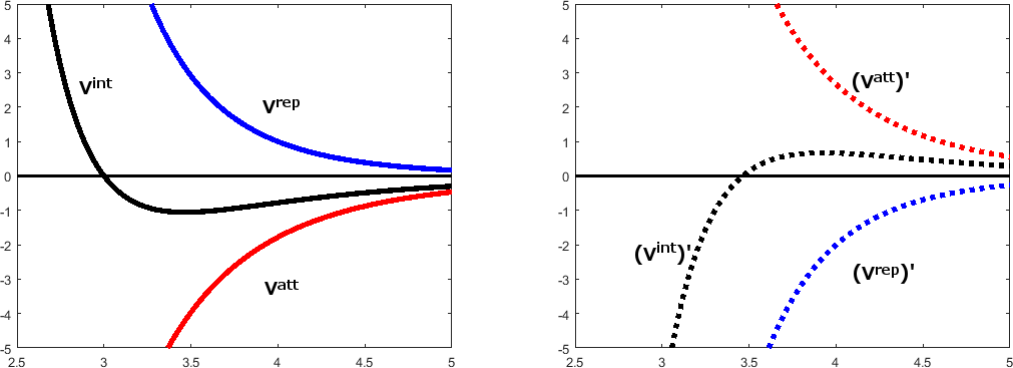
Left: The graphs of an interaction potential *V*^int^ composed of an attractive component *V*^att^ and a repulsive component *V*^rep^. Right: The corresponding slope functions.

PEFs *V *^int^(*r*) describing realistic molecular interactions, also called effective potentials, are always a sum of elementary attractive and repulsive components, *V *^int^ = *V *^rep^ + *V *^att^, not all of them need be true potentials. In general, effective potentials have a local minimum at *r*_equ_ and are, accordingly, not monotonic, however, they always have a repulsive branch left of the local minimum and an attractive branch right of it. Furthermore, they obey the asymptotic boundary condition. Examples are the Lennard-Jones potential or the Morse potential, see Figure 1. Because of the asymptotic boundary conditions, the constant interaction energy for large *r* is chosen as zero. Any system geometry **R**^diss^ with *V *^int^(**R**^diss^) = 0 represents the dissociated system, and the energy difference Δ*V* = *V *^int^(**R**^diss^) − *V *^int^(**R**^equ^) = −*V *^int^(**R**^equ^) is the adhesion energy.

If the potential depends only on the distance *r* between the particles, *V *^int^ = *V *^int^(*r*), the internal forces are central forces and automatically obey Newton’s third law. The first derivative or the slope function of *V *^int^(*r*) is the negative force function, *−F*(*r*) = [*V *^int^(*r*)]′. In this paper, we will always show PEFs together with their first derivatives instead of the force functions:


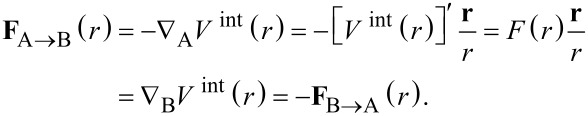


Because the interaction potential is the sum of attractive and repulsive components, the same is true for the internal forces, which are the sum of attractive and repulsive components, *F*(*r*) = −[*V *^int^(*r*)]′ = −[*V *^rep^(*r*)]′*V *^rep^ − [*V *^att^(*r*)]′. The first derivatives of the components are also monotonic and they obey the asymptotic boundary conditions.

For the description of interaction between subsystems, the forces corresponding to the attractive and the repulsive branch of the interaction potential are more important than the force components. For all distances *r < r*_min_, that is for the repulsive branch of *V *^int^(*r*), the force function has positive values, *F*(*r*) *>* 0 and the internal forces are repulsive. For the distances of the attractive branch, *r > r*_min_, the force function has negative values, *F*(*r*) *<* 0, and the internal forces are attractive. The attractive branch of the interaction potential *V *^int^(*r*) has an inflection point at *r*_infl_, where the slope function has a maximum. The maximum internal force is equal to the negative slope at the inflection point, *F*_max_ = *F*(*r*_infl_). In a complex of interacting molecular subsystems, attractive internal forces are called adhesive forces. At the local minimum of *V *^int^(*r*) the force function *F*(*r*) has a zero because the non-zero repulsive and attractive components of the internal force are equal in magnitude and, therefore, cancel out each other. For large distances *r*, that is for the dissociation of the system, the internal forces become zero because both force components become zero.

**Figure 2:**
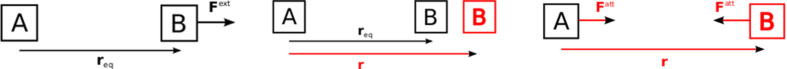
From left to right: An external pulling force acting on the system in its equilibrium structure increases the distance between the subsystems and induces an attractive internal force.

To separate subsystems from each other, an external force, called a pull-off force, must act on a subsystem and pull it off the other one. The point at which a pull-off force acts on the subsystem is called the pull-off point. External forces do not necessarily occur in pairs; thus, they are not genuinely balanced. Whenever a pull-off force acts on a system in its equilibrium the latter responds by inducing a pair of adhesive forces, see Figure 2.

**Figure 3:**
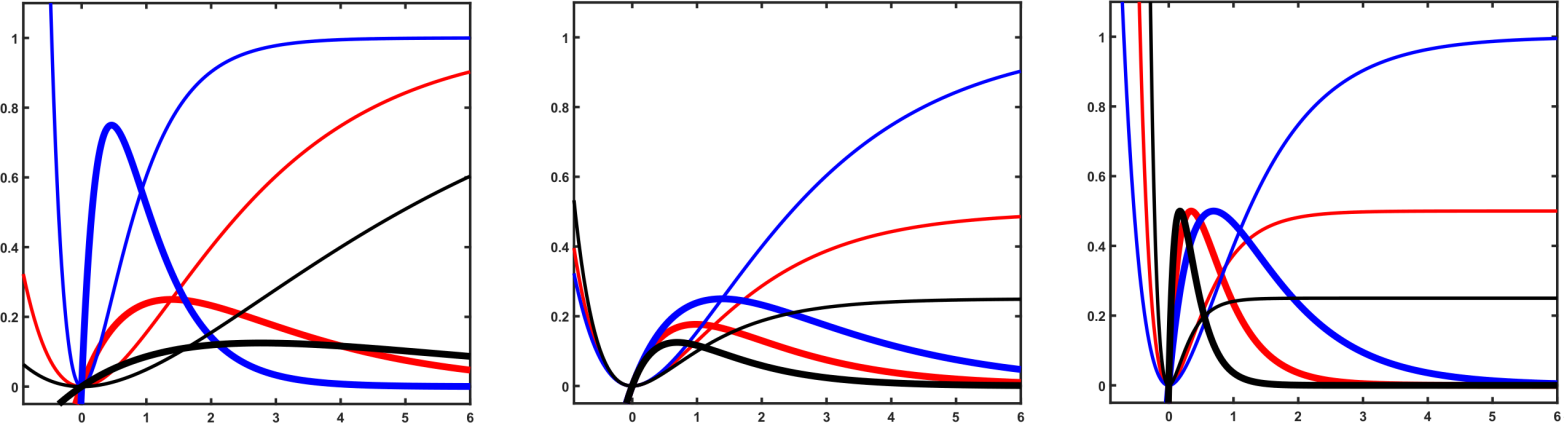
Potential functions (thin lines) and the first derivatives (thick lines). Left: For constant Δ*V* the maximum force decreases with decreasing curvature at the minimum. High curvature (blue), middle curvature (red), low curvature (black). Middle: For constant curvature the maximum force decreases with decreasing Δ*V*. Large Δ*V* (blue), middle Δ*V* (red), small Δ*V* (black). Right: Combination of large Δ*V* and small curvature may yield the same maximum force as small Δ*V* and large curvature.

Hence, both an external and an internal force act on the pull-off point, but in opposite directions. Stretching stops as soon as the adhesive forces are equal in magnitude to the pull-off force. Then, the pull-off force and the adhesive force are balanced and the system is in a new, stretched equilibrium structure. However, if the external force is larger in magnitude than the maximum adhesive force, the system dissociates and there is no stabilizing adhesive force. The maximum adhesive force therefore provides another measure of the system stability, which may differ considerably from that using the stabilization energy, Δ*V*. After all, *F*_max_ depends not only on Δ*V*, but also on the curvature of the potential curve at the minimum, see Figure 3. Therefore, interaction potential curves with the same Δ*V* can have different **F**_max_, or as Israelachvili says: “.. a bond may have a high bond energy, but a low force needed to break it. Thus, simply talking about the ‘strength’ of a bond may not mean anything” [6].

### Theoretical methods for the description of weak molecular interaction

Interactions in molecular system cause spatial displacements in the subsystems due to changes of the geometries and changes of the electron distributions. These intramolecular effects will, in turn, influence the intermolecular interactions between the distorted subsystems. Any interaction in a molecular system is the sum of intermolecular and intramolecular interactions. Molecules that strongly resist geometric distortions are called rigid or stiff, the resistance of electron distributions against distortion is called its hardness. By freezing the geometries of the interacting subsystems the interaction energy is calculated as if the interacting subsystems were ideally rigid. The intermolecular contributions to the interaction energy can be calculated in different ways.

In the supermolecule approach, the interacting system is treated as a large molecule and the stabilization energy is simply the difference between the energy of supermolecule *E*^AB^ and the sum of the energies of the isolated molecules *E*^A^ and *E*^B^:

[1]
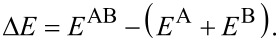


The mutual deformation of the electron distributions of the interacting molecules in the supermolecule is caused by attractive electrostatic interaction between electrons and nuclei, by mutual repulsion of electrons due to the charge, called Coulomb correlation, and by the mutual influence of the electrons due to the spin, called Fermi correlation. The advantage of high-level electron-structure methods is that they cover all these contributions and that they allow one to calculate weak molecular interactions for large distances between the interacting molecules, as well as strong molecular interactions when the molecules come very close. The disadvantage is that they are costly and, to explain the physical origin of the interaction energy, one has to split up the energy difference into physically meaningful contributions, which cannot be done in a unique way.

An alternative way of calculating the interaction energy is to make a multipole expansion of the interaction potential *V*_AB_(**r**_A_, **r**_B_) for the supermolecule and to calculate the energy contributions using perturbation theory:

[2]
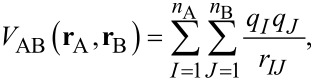


where *r_IJ_* is the distance between particles *I* and *J*. *V*_AB_(**r**_A_, **r**_B_) represents the interaction between the charge distributions of molecules A and B due to both the nuclei and the electrons. The nuclei are assumed to be point charges in space whereas the electrons form a continuous charge distribution. According to classical electrostatics, this gives rise to two basic contributions, called electrostatics and induction, also called polarization. However, there are purely quantum theoretical contributions to the interaction energy, both have to do with electron correlation. These two basic interactions are called dispersion interaction and exchange repulsion. With this approach, the interaction energy can be calculated at different orders, for all contributions a physical interpretation can be given. An obvious disadvantage of this approach is, however, that the multipole expansion can be done in different ways, and that the multipole terms have singularities when the distance between the expansion centers goes to zero.

### Basic interactions

#### Range of interactions

For interactions that only depend on the distance *r* between the interacting particles, and that can be represented by discontinuous model potentials, one can define the range of the interaction as the length of the interval of *r* values for which the interaction energy is negative. This definition is convenient for hard-sphere model potentials with a rectangular potential well, but it is less useful for continuous interaction potentials only going to zero for infinite distances. When the definition of range is based on forces, the mentioned hard sphere potentials are less useful because the derivative of such a potential is non-zero only at the discontinuities; that is, at the borders of the intervals where the interaction energy is negative. Anywhere else, the forces are zero. For continuous potentials, one can define the range as the length of the interval for which the potential or the force is significantly larger than zero. Using the extension of the Yukawa potential to general screened potentials, 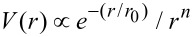, with a power *n* ≥ 0, allows interactions to be classified as being of infinite range when *r*_0_→∞, otherwise they have the finite range *r*_0_. According to this definition, all potentials depending on powers of the inverse distance, e.g., all electrostatic, induction and dispersion interactions, are of infinite range, whereas exchange repulsion is of finite range. For all functions of infinite range, the power *n* can be used to distinguish between shorter and longer ranges: the smaller *n*, the longer the range. Another caveat by Israelachvili is the following: “It is […] wrong to associate long-range *effects* with long-range *forces*. In fact, the opposite is usually the case – for what is more important is the strength of the interaction, and […] short-range forces tend to be stronger than long-range forces” [6].

#### Exchange repulsion

Exchange repulsion, or Pauli repulsion, is a consequence of the Pauli exclusion principle, which states that Fermions avoid coming spatially close to each other. Thus, exchange repulsion has an enormous impact on the spatial distribution of electrons in molecular systems. The effect of keeping electrons at a distance “plays the role of a fictitious, although highly effective, mutual repulsion being exerted within the system, irrespective of any other actual forces of interactions […] that might be present” [16]. Exchange repulsion is a nonlocal effect of purely quantum origin, it is ubiquitous and it is fundamental. As Lennard-Jones wrote 1954: “This effect is most powerful, much more powerful than that of electrostatic forces. It does more to determine the shapes and properties of molecules than any other single factor. It is the exclusion principle which plays the dominant role in chemistry” [17]. Exchange repulsion can be described by a repulsive potential–energy function with exponentially decaying dependence on the interatomic distance [18–19]. Its representation by a potential–energy function is similar to the use of local, repulsive “pseudo”-potentials. The assumption that exchange repulsion between any two molecules can be represented by a single exponential is not justified, there need to be more.

#### Electrostatics

Electrostatics is the classical interaction between static electric multipoles, which are obtained by a multipole expansion of the charge distribution of a molecule about a convenient expansion point, usually the center of mass. Static multipoles 2*^l^* of rank *l* = 0, 1, 2,… are monopoles (*l* = 0), dipoles (*l* = 1), quadrupoles (*l* = 2), and so on. The interaction potential for the interaction between an *l*-pole and an *L*-pole has a distance dependence of 1/*r^l+L+1^*. The higher the the multipoles, the shorter the range of interaction. The Coulomb interaction, i.e., the interaction between electric monopoles, that is, charges, has the longest range. The interaction between static multipoles may be attractive or repulsive. The sign of the Coulomb interaction depends only on the signs of the charges. If at least one higher multipole is involved, the interaction also depends on the relative orientation of the multipoles, meaning that it can be attractive, repulsive or that there is no interaction at all. The interaction potential between an *l*-pole and an *L*-pole can be quite generally written as

[3]
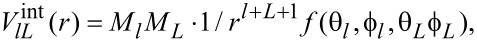


where *M_l_* and *M_L_* are the magnitudes of the *l*-pole and the *L*-pole, and 
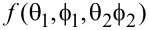
 is the geometric factor describing the relative orientation of the two multipoles with respect to the line connecting the centers of mass using local spherical polar coordinates. The product of the magnitudes of the multipoles is often used as a measure of the strength of interaction, which is modulated by the angular dependence of the geometric factor. The product *M_l_M_L_*·1/*r^l+L+1^* must have the physical dimension energy, the geometric factor is a bare number. The strength of Coulomb interaction is proportional to the product of the charges.

Although any spatial charge distribution can be expanded into a series of multipoles, the number of terms can be large when the symmetry of the charge distribution is low. One can avoid working with high-rank multipoles when the single-center expansion is replaced by a multicenter expansion, also called a distributed multipole expansion, in which several meaningful expansion centers are chosen, for example the positions of the nuclei in a molecule or the centers of mass of atom groups. Each expansion then contains only few multipoles. Regardless of whether single-center or multicenter expansions are used, the electrostatic interaction energy becomes singular only when the distances between the expansion points become zero. For extended charge distributions, the multipole expansion of the electrostatic interaction energy is in error as soon as the charge distributions overlap. Classical electrostatics shows that the interaction energy for extended charge distributions is much smaller in magnitude than that of point-multipoles. Correctly calculated electrostatic interaction energies do not have singularities. The difference between the interaction energy for extended charge distributions and the multipole expansion is called the penetration error. It can be corrected either by using damping functions or by applying a penetration error correction [20]. However, one should not overestimate the physical significance of this correction, the overlap of hard electron distributions is purely fictional, after all, both Fermi and Coulomb interactions are not considered.

#### Induction

The second class of classical interactions covers those between the static electric multipoles in molecule A and the induced multipoles in molecule B. The latter are the result of charge shifts (polarization) in the polarizable electron distribution of molecule B. The strength of the interaction is measured by the respective static polarizabilities, which describe the ability of polarizable systems to create induced multipoles under the influence of nonuniform electric fields. The interaction between a static *l*-pole and an induced *L*-pole has a 1/*r^2(l+L+1)^* distance dependence, and again it depends on the relative orientation of the multipoles. Induction interactions are therefore always of much shorter range than the interactions between the corresponding static multipoles. The polarizability that describes the interaction between a static and an induced dipole is called the dipole–dipole polarizability. Likewise, for the interaction between a static dipole and an induced quadrupole the dipole–quadrupole polarizability is responsible, and so on. The interaction with the longest range is again the dipole–dipole interaction. However, at short distances, e.g., the equilibrium distance, the short-range interactions become important. Unfortunately, the corresponding polarizabilities are seldom tabulated. After all, they are tensor quantities, and one cannot infer from tabulated dipole–dipole polarizabilities whether or not the higher short-range interactions are important or not. As for electrostatic interactions, the induction energy at short distances between the multipoles is in error for point-multipoles, but can be corrected when damping functions are used [2].

#### Dispersion

The dispersion interaction is a ubiquitous interaction of purely quantum origin. It is a kind of dynamic electron correlation, and between ground-state molecules it is always attractive. Its description is far from simple [21]. A catchy albeit disputable explanation starts with short-time deformations of the electron density of one molecule caused by the non-deterministic motion of electrons. These fluctuations are represented by spontaneously created multipoles that will interact with induced multipoles in the electron distribution of the other molecule. Any nonsymmetric deformation leads at least to a dipole. The induced multipole of lowest rank in the other molecule is again a dipole. The 1/*r*^6^ distance dependence of the corresponding dipole–dipole dispersion interaction again has the longest range and is the leading contribution at large distances. At shorter distances, higher-order dispersion interactions of much shorter range are again important. For two interacting atoms, the interaction energy is isotropic because it depends only on the interatomic distance. For molecules, an effective isotropic dispersion interaction follows from averaging over all relative orientations of the multipoles. The distance dependence of the dispersion interactions is the same as that of the corresponding induction interactions. The strength of the interaction between atoms is proportional to the product of the dynamic polarizabilities [2]. It is much larger between noble-gas atoms from the higher periods, than between atoms with hard electron densities [22–23]. For molecules, one has to consider the anisotropy of molecular polarizabilities, which is strongly pronounced for molecules with delocalized pi-electron densities, the polarizability component along the molecular axis, that is the polarizability of the p-electrons, is always considerably larger than the components orthogonal to it [24–25]. Dispersion energies calculated with this method at short distances between the multipoles are in error. Again, damping functions help to avoid these errors.

Note, that the 1/*r*^6^ distance dependence does not hold for atoms or small molecules interacting with extended metals or perfect graphene, for such systems one finds a 1/*r*^3^ distance dependence [22–24].

### Combination of the basic interactions

For non-charged systems with spherical electron distribution (atoms), there are no electrostatic or induction interactions. There are only dispersion interactions, starting with the long-range dipole–dipole interaction. This interaction exists between any two molecular systems. Every non-charged and non-spherical molecule has static multipoles of different ranks, in polar molecules, the series starts with dipoles, whereas in non-polar molecules it starts with quadrupoles or higher multipoles. Accordingly, there will always be electrostatic interactions of different ranges between molecules with static multipoles. For example, the T-shaped equilibrium structure of the benzene dimer is favored by the geometric factor of the quadrupole–quadrupole interaction [2]. Every molecular system has a polarizable electron distribution, in which multipoles can be induced. Therefore, if at least one subsystem has static multipoles of any rank, there will be induction interactions.

If we combine the products of the magnitudes of the multipoles and the geometric factors to prefactors *P_n_*, the interaction potential for two uncharged molecules can be written as a series

[4] $$\begin{array}{c} V^{\text{int}}\left(r\right)=V^{\text{exc}}\left(r\right)+V^{\text{elst}}\left(r\right)+V^{\text{ind}}\left(r\right)+V^{\text{disp}}\left(r\right)\\ =\sum_{i}P_{i}^{\text{exc}}e^{-a_{i}r}+\sum_{n=3}^{\infty }P_{n}^{\text{elst}}\frac{1}{r^{n}}\\ +\sum_{n=6}^{\infty }P_{n}^{\text{ind}}\frac{1}{r^{n}}+\sum_{n=6}^{\infty }P_{n}^{\text{disp}}\frac{1}{r^{n}}. \end{array}$$

The number of terms in the series that contribute significantly depends on the magnitude of the corresponding prefactors and also defines the “flavor” of the interaction.

### Many-body effects

Many-body systems [25] are composed of particles of different kind. Each particle interacts with all others, that is, all particles are highly correlated, otherwise one would have many one-body systems. The nature of the particles depends on how the systems is modeled. In an electron gas the particles will be electrons. If an atom is regarded as a many-electron system, they will be electrons. If a molecule is regarded as being composed of atoms, the particles will be atoms, but if the molecule is modeled as a many-electron system, the particles will be electrons again. In a liquid or a molecular crystal, the particles may be molecules, they may be the atoms or they may be electrons. Frequently, many-body systems behave as if the particles interact only weakly or do not interact at all. But these particles are not the real, strongly interacting particles but fictitious particles, called quasi-particles. Calculating the energy of the many-body system by summing up the interactions between all real particles is impossible. Weak interactions between quasi-particles can, however, be calculated using conventional techniques, e.g., perturbation methods. A simple introduction to the idea of quasi-particles goes as follows: All interacting particles are in motion, so any particle may interact with two or more other particles at the same time, and a certain interaction may occur repeatedly in a certain time interval. A strategy for defining quasi-particles is to identify and keep a few of the most important interaction types between the real particles and to neglect all others. Furthermore, it is assumed that it is easy to calculate the sum of all repeated occurrences of these interactions. By adding these partial sums of interactions with the other particles to the real particle it is transformed into a quasi-particle. Some properties of quasi-particles may be different from those of real particles, others are unchanged. A good introduction to quasi-particles in many-body systems can be found in the book by Mattuck [26]. Depending on what kind of real particle is transformed into a quasi-particle, different properties are of interest and different theoretical quantities are used to describe them. For example, electrons as described by Hartree orbitals, Hartree–Fock orbitals or Kohn–Sham orbitals are quasi-particles. They exhibit a different extent of interaction with other electrons, they have different (orbital) energies but the charge is not changed. (Quasi)-atoms in a molecule have, for example, volumes and polarizabilities that differ from those of free atoms in the gas phase. Properties of such quasi-atoms are often calculated by using propagators (Green’s functions) or response functions that were obtained by using a special summation of important interactions, for example by using the random phase approximation. The weak interactions not absorbed into quasi-particles are calculated as interactions between quasi-particles. The magnitude of these interactions depends essentially on the way the quasi-particles are created. Frequently, it is assumed that the weak interactions are dominated by pair contributions, and that interaction between three or more quasi-particles can be reduced to sums of pair interactions (additivity of interaction). Whether or not this assumption is justified depends on the many-body system, and on the extent to which the interaction between the real particles is included in the quasi-particles.

Many dispersion-correction strategies assume pair-wise additivity of the long-range electron correlation energy. The properties of the quasi-atoms may be obtained by fitting them to interaction energies calculated with other high-level methods. This strategy is used, for example, in Grimme’s D2 method [27], for the calculation of the dispersion energy in the dlDF+D method by Szalewicz [28–29], and for the dispersion correction to DFTB [30–33]. In the Tkatchenko and Scheffler (TS) method [34], the *C*_6_ coefficients for atoms in a molecule are set proportional to those of the corresponding free atoms. The proportional constant is a function of the ratio between the volume of the free atom and the Hirshfeld volume of the atom in the molecule. According to Dobson [35], one can distinguish between three different types of non-additivity of dispersion interactions. Type-A non-additivity originates from the fact that the dispersion coefficients of free atoms are different from those of atoms in molecules. This type of non-additivity is captured for example by the TS model and Grimme’s D3 method [36] by employing environment-dependent dispersion coefficients. Type-B non-additivity occurs, when the interaction between two particles is screened by a third particle, giving a three-center angularly dependent interaction contribution. The most simple three-body term is a triple–dipole contribution, the so called Axilrod–Teller–Muto term, which, because of the angular dependence, can give attractive and repulsive contributions. This three-body correction is included in Grimme’s D3 method. When *N* perturbing particles are considered, one gets *N*-center contributions. In diagrammatic many-body theory, interactions of that kind are represented by ring diagrams [26], summation of ring diagrams to infinite order gives the correlation energy in the random phase approximation. Type-C effects, according to Dobson’s classification, can be found in nanostructures of low dimensionality with degenerate electronic ground states where any perturbation causes delocalized density fluctuations or density waves, also called collective excitations [25–26]. Often they are found in one- or two-dimensional structures such as graphene or metallic nanotubes with easily polarizable electron densities, and they are less frequently found in three-dimensional metals [35,37–38]. Delocalized density fluctuations allow for the induction of large dipoles or higher multipoles that enhance weak molecular interaction: It is characteristic of interactions between such extended density waves that the range of the interactions is much longer than that of dispersion interactions between localized structures [35]. Dispersion interactions are a type of electron correlation, but dispersion interaction is not a synonym for electron correlation. Therefore it is clear that there must be other types of electron correlation beyond dispersion interaction. It is also clear that there are many different types of collective motions in extended systems [25]. It should not be surprising that interactions between different density fluctuations may have different ranges.

Different strategies can be used for improvement of the description in many-body systems. One is to go beyond the triple-dipole term in the calculation of three-body energies, formulas for the dipole–dipole–quadrupole or dipole–quadrupole–quadrupole terms are given, e.g., in the book by Salam [21]. Due to the distance dependence of these terms they are only significant at short range, and they are strongly anisotropic [2]. Another strategy is to keep the description of the interacting atoms in molecular systems as simple as possible but to include the interaction between many of these atoms. This route is followed in the many-body-dispersion (MBD) method by Tkatchenko and co-workers [34,39–40]. The atoms are considered to be isotropic, oscillating charge distributions represented by 3D harmonic oscillators, the polarizabilities are obtained with the TS method. Interaction between the atoms considered as vibrating dipoles yields screened atomic polarizabilities that are finally used to calculate long-range correlation energies from diagonalizing the Hamiltonian of the coupled oscillators with the screened polarizabilities as input. Although by this procedure many-body contributions are captured that go beyond the three-body ATM term and improve, for example, cohesive energies considerably [37,41], some open questions concerning the calculation of correlation energies using the MBD method remain. For example, it is not yet clear how well fluctuating dipoles represent fluctuations of anisotropic charge in general, or whether molecular polarizabilities entering the expressions for dispersion interaction in the single-center expansion can be replaced by fragment polarizabilities, analogous to the multi-center expansion of charge distributions [38].

With respect to the calculation of adhesive forces, no detailed MBD studies are available, especially it is not clear, how strong the many-body effects change the shape and slope of the adhesion energy curves around the inflection point.

### Range of electrostatic interactions

Electrostatic potentials of 2*^l^*-poles depend on the distance according to 1/*r^l+1^*, and the electric fields depend according to 1/*r^l+2^*. High-rank multipoles can be approximately represented by multipoles of lower rank at different spatial positions, i.e., a dipole can be represented by two charges (monopoles), a quadrupole by two dipoles or four monopoles, and so on. But when this is done, one must not forget the correct distance dependence of the high-rank multipole–multipole interaction. Since the interaction between an *l*-pole and an *L*-pole is proportional to 1/*r^l+L+1^*, the interaction between a dipole and a charge is proportional to 1/*r*^2^. If this is ignored, one could believe that there is a Coulomb interaction between monopoles, which has, however, a 1/*r* distance dependence. That the field of spatially close charges has a different distance dependence than isolated charges far apart shows the electrostatic potential of an ionic crystal, which is composed of a large number of monopoles. The interaction between a test charge and, e.g., a rock-salt crystal, operates at very short distance, and not at distances as large as one might assume, considering the long range of Coulomb interactions. However, close to each charge in the crystal, there is a charge of opposite sign forming a dipole with a field that is proportional to 1/*r*^2^. Close to each dipole is another dipole and the resulting quadrupole field is proportional to 1/*r*^3^. Two quadrupoles close to each other form an octopole with a 1/*r*^4^ distance dependence, and so on. This means that the potential of an ionic lattice decays faster with *r* than any power of 1/*r*, which means an exponential decay. The finite range of such a potential is smaller than the spacing between the ions in the crystal [6]. Elementary classical electrostatics shows, thus, that superpositions of low-rank multipoles with large range located at different positions in space are equivalent to high-rank multipoles with a much shorter range. But this is frequently ignored in chemistry, where, for example, interactions between two molecular quadrupoles (1/*r*^5^ distance dependence) are reduced to interactions between bond dipoles having a 1/*r*^3^ distance dependence.

### Contact zone

Interactions between atoms or finite molecules are dominated by pair contributions, even when many-body contributions are shown to be important, as, for example, in the case of the non-additive induction interaction [2]. We will now consider the pair contributions to the long-range dipole–dipole dispersion interactions between molecules A and B with *n*_A_ and *n*_B_ atoms, respectively, which are used to define the contact zone (CZ) of atoms or interacting molecules:

[5]
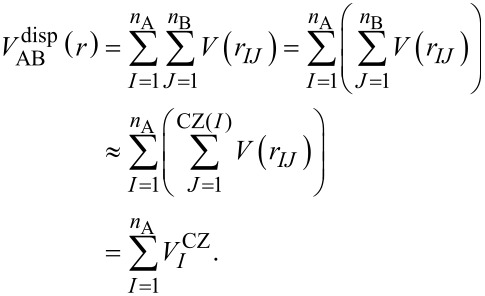


For atom B*_J_* in molecule B nearest to atom A*_I_* in molecule A with pair distance *r* = *r_IJ_*, the pair contribution to the dipole–dipole dispersion interaction is proportional to 1/*r*^6^. For all atoms B*_J_* with a pair distance larger by a factor of *f* with *f >* 1, the pair contribution is reduced by 1/*f*^6^. Due to the sixth power, the magnitude of the pair contribution decreases strongly with increasing distance: When *r* increases by 10%, the pair contribution is reduced by 44%; when *r* increases by 50%, the pair contribution is reduced by 91%. The contributions to the attractive short-range dispersion drop even faster, as do the contributions to the exchange repulsion. For the 1/*r*^12^ term in the Lennard-Jones potential, if *r* increases by 20%, the interaction is reduced by 89%. Therefore, each atom in molecule A will “see” only few atoms from molecule B; the others can safely be neglected. Dispersion interactions, induction interactions and exchange repulsion are “near-sighted”, as are electrostatic interactions between high multipoles. Whenever *f* ≥ 1.5, that is, when the pair distance *r* is more than 50% larger than the equilibrium distance *r*_equ_, the contributions will be close to zero. From here on, we will always speak of a distance *r* = 1.5*r*_equ_ as the threshold value. All atoms B*_J_* that give non-negligible contributions to the interactions with atom A*_I_* make the contact zone CZ(*I*) of atom A*_I_* in molecule B. The sum over *J* in Equation 5 can thus be limited to the atoms in the CZ causing only a small and acceptable loss of accuracy. The sum over atoms *I* shows that the CZs are approximately additive as a consequence of the “near-sightedness” of WMI.

The concept of near-sightedness of electrons was introduced by Kohn [42] in the description of many-atom systems, and “[i]t can be viewed as underlying such important ideas as Pauling’s ‘chemical bond’, ‘transferability’…” [43], about which Prodan and Kohn say: “Understanding the physics and chemistry of large molecules and solids would have been practically impossible if not for the principle of transferability” [43]. In the language of density functional theory, the concept of near-sightedness of electrons “…describes the fact that, for fixed chemical potential, local electronic properties, such as the density *n*(*r*), depend significantly on the effective external potential only at nearby points. Changes of that potential, no matter how large, beyond a distance *R* have limited effects on local electronic properties, which rapidly tend to zero as a function of *R*” [43]. In their 2005 paper, Prodan and Kohn list what near-sightedness of electronic matter is not. For example, it is not screening of charges, as it applies also to neutral fermions, it “does not apply to systems of few electrons” and “it is not limited to macroscopically homogeneous systems” [43]. We explain the approximate additivity of dispersion interactions between molecular systems by a similar near-sightedness of WMI, caused by the short range of the basic interactions. The concept of near-sightedness of WMI is not the same as the near-sightedness of electrons, the distance *R* mentioned by Prodan and Kohn is different from our threshold value described above. Near-sightedness of electronic matter is of finite range, it explains why linear scaling in electronic structure methods works. The near-sightedness of the attractive basic interactions in WMI, on the other hand, is of infinite range, but it allows to understand the transferability of group contributions of, for example, pairs of CH_2_ in two parallel aligned alkane chains. For the interaction of atoms or small molecules with extended metal surfaces, the concept of near-sightedness of dispersion does not apply, because the polarization of the metal due to the small interaction partner is not local, there are collective polarizations in the metal, rather than local ones [22–23].

**Figure 4:**
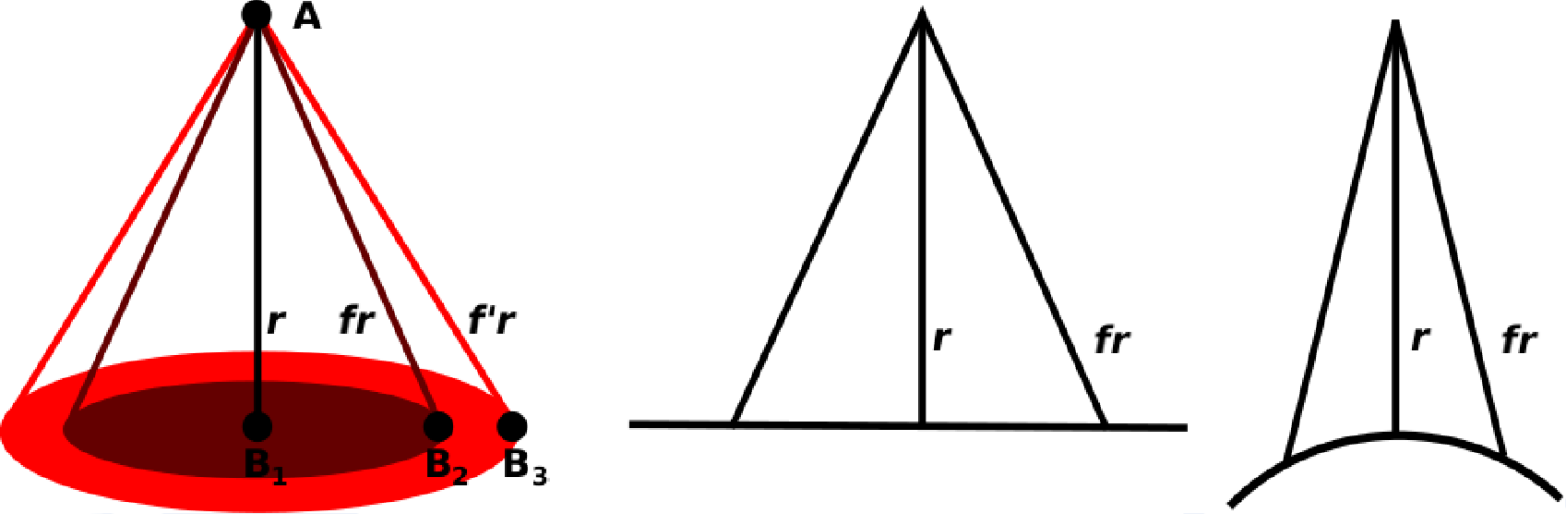
Left: The disk covering the atoms of molecule B seen by an atom in molecule A expands with increasing lateral height *fr* of the “vision cone”. Right: The aperture of the vision cone becomes smaller when molecule B is not planar but bent.

For an atom, the shape of its CZ in a planar molecule is a disk that is the base of a cone with a lateral surface composed entirely of lines of length *fr*, which is the “vision cone” of the atom, see Figure 4. The disk contains all atoms B*_J_* that make a contribution larger than 1/*f*^6^. If atom A*_I_* interacts with atoms B*_J_* of a curved molecule B, say a fullerene or a carbon nanotube, the CZ is smaller than when the molecule is planar. See the right-hand side of Figure 4. Of course, one can do the same with the roles of molecules A and B reversed. Therefore, the CZ of two interacting molecules can be defined as the set of all atom pairs contributing significantly to the interaction energy. This is in accord with the success of distributed multipole expansions of all basic interactions.

**Figure 5:**
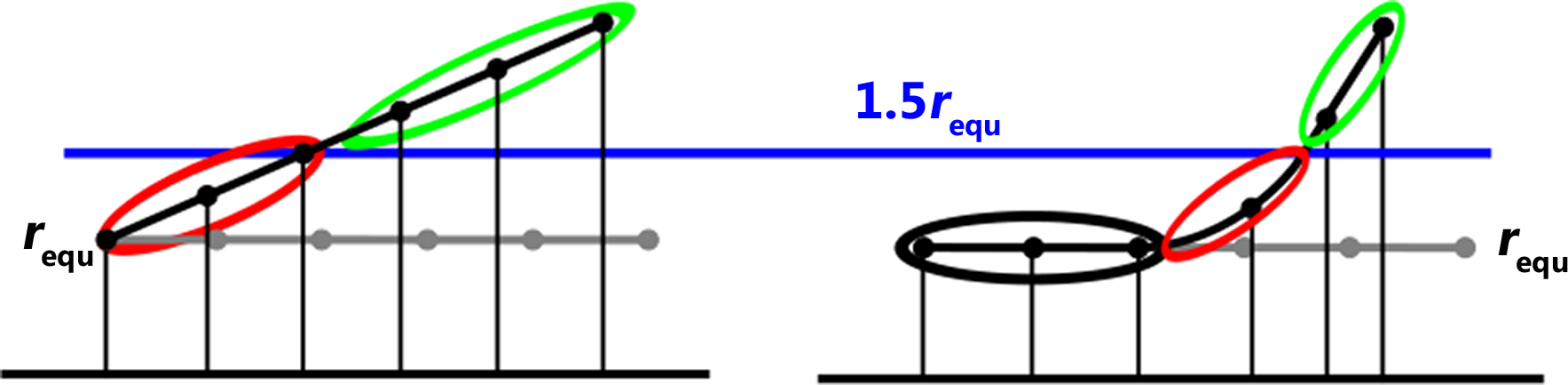
Demonstration of the contact zone and the reduced contact zone of an adsorbate/adsorbent complex with equilibrium distance *r*_equ_ during separation in modes S2 and S3. Left: In separation mode S2, all atom pairs, except the one with the pivot atom, change their distance *r_IJ_*. Atom pairs with distances larger than the threshold value 1.5*r*_equ_ (adsorbate atoms encircled in green) do not contribute to the stabilization energy or to the adhesive forces. Non-zero contributions come from atom pairs in the reduced contact zone (adsorbate atoms encircled in red). Right: In separation mode S3, some part of the contact zone remains unchanged (adsorbate atoms encircled in black). These atom pairs contribute most to the stabilization energy, but not at all to the adhesive forces. The atom pairs with distances larger than the threshold value (adsorbate atoms encircled in green) do not contribute to the stabilization. Atom pairs in the reduced contact zone (adsorbate atoms encircled in red) contribute less to the stabilization energy than atom pairs from the (black) contact zone but they are the origin of the change in the adhesive forces.

The maximum interaction energy is proportional to the size of the CZ at the equilibrium geometry of the complex. Any decrease in the size of the CZ brought about by increasing the distance between the interacting molecules reduces the interaction energy and reduces the adhesive forces in the complex. The change in the interaction energy, and therefore the magnitude of the adhesive forces, is proportional to the changing part of the CZ where the pair distances *r_IJ_* increase and the adhesion energy decreases; this is the reduced contact zone [44], see Figure 5.

### Other representations of basic interactions

A problem in speaking about WMI is that, in chemistry, often a stinted and frequently unphysical language is used. Although electrostatics and induction have very different ranges, induction is often, incorrectly, included under electrostatics, rather than being separately discussed. If induction is considered, it is described in terms of the dominant theoretical means mastered by chemists, namely orbitals. Polarization of the electron distribution of atoms manifests itself, for example, in an increase of the weight of the polarization functions in the occupied atomic orbitals (AOs). This could be shown, for instance, by adding p-type or d-type basis functions to occupied AOs having purely s-character in the unpolarized atom. This is nothing else than the hybridization of AOs. The molecular orbitals (MOs) of a complex of non-interacting molecules are, in general, linear combinations of the occupied fragment MOs, that is, the MOs of the isolated molecules. If such a complex MO is dominated by a fragment MO of one molecule, the complex MO is localized on that molecule. Induction or polarization will change the weights of the fragment MOs in the complex MOs. Localized complex MOs may then become delocalized, which is frequently called by chemists “charge transfer”, and it is claimed that the charge-transfer interaction is an important, stabilizing interaction. Charge transfer, however, refers to an ion pair stabilized by a strong Coulomb interaction with a much larger stabilization energy than that of a weakly interacting system. Describing a charge shift in the electron density of a molecular system as a charge transfer incorrectly twists the semantics of the word transfer.

Quantum theory says that states of subsystems may interfere whenever the subsystem wave functions overlap significantly. Because the wave functions of atoms or molecules decay exponentially, this only happens at short distances between the subsystems. Ruedenberg et al. [45–51] showed that covalent bonding is a one-electron effect, and that the so-called accumulation of charge between the atoms connected by a covalent bond is a charge shift caused by constructive interference of exponentially decaying AOs or hybrid AOs. Thus, covalent bonding operates only at much shorter distances than those between weakly interacting molecules. At distances as large as those between weakly interacting molecules, the overlap of the molecular wave functions and the ensuing stabilization are very small, given that there is indeed constructive (and not destructive) interference of the many-electron state functions of the interacting molecules. Nevertheless, it is frequently claimed, but not proven, that strong covalent bonding is important for hydrogen bonding.

Rather curious are so-called orbital–orbital interactions such as π–π interactions, because orbitals are one-electron state functions, which do not interact but may be used to describe interacting states. However, it is never quite clear what kind of “interactions” they are describing. Are they describing static attractive multipole–multipole interactions between orbital contributions to the molecular electron densities, as Anthony Stone suggests [2]; or are they describing constructive or destructive interference of orbitals similarly as for the explanation of reactions using the Woodward–Hoffmann rules? Are they describing attractive dispersion interactions between the π-densities, or the exchange repulsion of π-densities?

### Methods to describe WMI

WMI stabilization energies for interacting molecules A and B are calculated either with the supermolecule method or with perturbation methods. In the supermolecule approach, the interacting complex is treated as a supermolecule and the stabilization energy is simply the difference between the energy of the supermolecule *E*^AB^ and the sum of the energies of the isolated molecules *E*^A^ and *E*^B^:

[6]
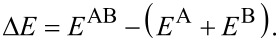


The energies can be calculated with any high-level electron structure method. The Hamiltonian of the supermolecule is

[7]
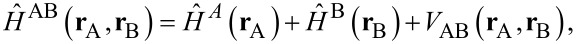


where 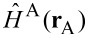 describes the isolated molecule A with particles *I* having position vectors **r**_I_ and charges *q_I_*. Analogously, 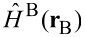 describes molecule B, and *V*_AB_(**r**_A_, **r**_B_) describes the Coulomb interaction between all particles of A with all particles of B:

[8]
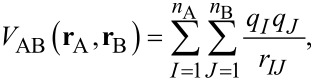


where *r_IJ_* is the distance between particle *I* and *J*. Whereas the geometry of the supermolecule is nearly always optimized, the isolated molecules may either be in their corresponding equilibrium geometries or in deformed geometries, depending on whether the interaction energy includes the deformation energies of the interacting molecules or not. A well-known problem with the supermolecule approach is the basis set superposition error (BSSE). Because of the finite one-particle basis, counterpoise corrections (CPC) are necessary to get reliable interaction energies.

In the perturbation approach, the unperturbed Hamiltonian for the complex is 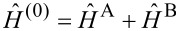. Here, the geometry of the interacting molecules determines the geometry of the complex. It is assumed that the ground- and excited-state functions 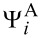 and 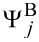 of the interacting molecules are known, the wave functions of the complex are then simply the products 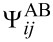 = 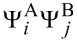, they are eigenfunctions of 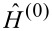. The energy of the interacting complex is the sum of the energy contributions of different order:

[9]
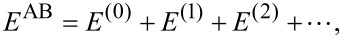


with

[10]
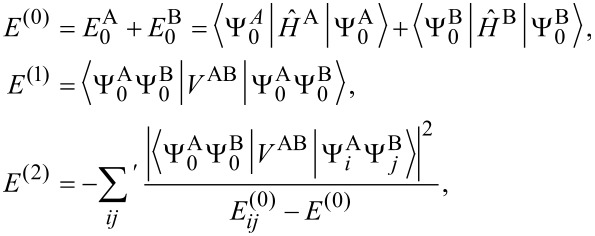


where 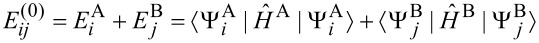 and the prime on the summation sign indicates that *i* and *j* are not zero at the same time.

Since all perturbation contributions are calculated by using the wave functions of the isolated molecules, there is no BSSE and no CPC is needed. The first-order correction *E*^(^*^1^*^)^ is simply the electrostatic interaction energy, whereas the second-order contributions are the sum of the induction and dispersion energies. This perturbation series is correct for interacting molecules far apart, because for them, the simple product 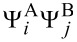 is an eigenfunction of 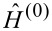. For shorter distances, the exchange of electrons between the two molecules must be considered, and the correct wave function for the interacting complex is 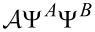 where 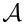 enforces the exchange of all electrons of A with those of B. But this wave function is no longer an eigenfunction of 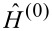. There are many perturbation approaches with correctly antisymmetrized wave functions. One of them is symmetry-adapted perturbation theory (SAPT) [52]. We use the variant in which the intramolecular energies 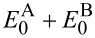 are calculated with density functional theory (DFT) and only the intermolecular contributions are calculated with perturbation theory. This approach is called SAPT(DFT) [53–54].

The electronic-structure methods used together with the supermolecule approach must be able to cover the largest part of electron correlation. Among wave-function methods, the coupled cluster method at the CCSD(T) level is currently the best method available. Conventional DFT methods cover short- and medium-range electron correlation, but not long-range correlation, which includes dispersion interactions. To correct this deficiency, empirical dispersion corrections were developed [27,34,36,39–40,55–57], which, when added to the DFT energy, yield energies of comparable quality to CCSD(T). This class of methods is called DFT+D: They are discussed in reviews such as those by Grimme and Tkatchenko [38,58]. Empirical dispersion corrections are often the sum of pair contributions calculated with strongly parameterized functions that depend only on the positions of the atom pairs, and are independent of the basis functions used with the electronic structure methods. Only the latter require CPC. For large systems, conventional DFT is often too costly and therefore semiempirical DFT methods such as DFTB (density functional tight binding) [30–31] are used, together with empirical dispersion corrections [32–33]. With these methods, intramolecular dispersion interactions in large molecules can be embraced.

If one is only interested in intermolecular dispersion corrections, one could directly calculate the pair contributions, instead of first calculating the dispersion contributions for each interacting molecule and the supermolecule, and then calculating the difference. This is the basis of the dlDF+D approach [28–29], with a dispersionless density functional that reproduces the CCSD(T) correlation energy of an interacting system without any dispersion contributions. The dispersion contributions to the interaction energy are calculated pairwise with a function that was fitted to SAPT(DFT) dispersion energies. The dlDF contributions are calculated using the supermolecule approach. These energies require CPC. All methods mentioned have been used in our studies on weakly interacting systems.

## Results

All investigations on adhesion of aromatic molecules onto carbon nanotubes and graphene presented here have been published [13–15,44]. All calculations were done with the DFTB+D method as implemented in the DFTB+ code [59]. In [13–14,44] periodic boundary conditions were used. In [15] all systems were treated as large clusters.

The description of system stabilization due to adhesion can be done by using either adhesion energies or adhesive forces. We use both concepts to describe stabilization of the systems investigated.

### Adhesion energies

The starting point for these investigations was the claim, that (8,0)-carbon nanotubes (CNT) can be dissolved in aniline [60–61]. If this was true, the CNT should be more strongly bound to the aniline molecules in the first solvation shell than to other CNTs in a bundle, to prohibit solvated CNTs from aggregating and eventually precipitating. However, this was found not to be the case [13]. The stabilization energy for a (8,0)-CNT tightly covered with an aniline monolayer was only 40% of that of a bundle of CNTs in which one CNT is hexagonally surrounded by six other CNTs. We used stabilization energies normalized to the unit length. We have pointed out [13] that two parallel CNTs in their equilibrium geometry have one CZ, while three parallel CNTs with their molecular axis lying in a plane have two CZs. Accordingly, in a complex of a CNT surrounded by six CNTs, that is, covered by a monolayer of CNTs, there are six CZs between the central CNT and the monolayer, but there are also six further CZs within the monolayer – altogether 12 CZs. We found that the stabilization is indeed twelve times larger than that of a CNT dimer with one CZ. The aniline monolayer was found to consist of six strips of aniline molecules, similar to the monolayer of six CNTs. However, the stabilization energies show the differences between the two systems: for two CNTs in contact, the stabilization energy (7.33 kJ/mol·Å) is about 20% larger than for an aniline strip in contact with a CNT (5.02 kJ/mol·Å). For a monolayer of aniline molecules, the stabilization energy per aniline strip is 6.08 kJ/mol·Å. The 20% increase is caused by the interaction between the six aniline strips touching each other at the edges. Likewise, for a CNT covered by six CNTs the interaction per CNT in the monolayer is 15.07 kJ/mol·Å. The 106% increase is caused by the interactions between the CNTs in the monolayer. Both increases reflect collective effects due to interactions between molecules forming the monolayer. They also show that the edge-to-edge interaction between aniline strips is much smaller than the face-to-face interaction between aromatic molecules. Therefore, it is not surprising that a complex of an aniline strip inserted between two CNTs is less stable than two CNTs in contact with each other and the aniline strip in contact with one CNT. Accordingly, it would be highly unfavorable for an aniline molecule to separate two CNT molecules and insert itself between them, as it would need to happen if aniline were indeed a solvent for solid CNT. Although aniline has a permanent dipole moment (1.56 D) slightly smaller than that of water (1.87 D), there is no significant difference in the stabilization energies of parallel and antiparallel orientations of two linearly arranged aniline molecules. In the complex of a CNT and an aniline strip there will be stabilizing contributions from the permanent aniline dipole and the induced CNT dipole. At the CZ of two parallel CNTs, there will be a stabilizing interaction between the permanent dipole moments originating in the curvature of the CNT molecules. Nonetheless, dispersion interactions are the major stabilizing contribution for both systems, and they are also the origin of the difference in the stabilization. In a strip of aniline molecules, there is a large distance between the phenyl rings caused by the CH bonds and the NH_2_ groups, and in this gap there are far fewer atoms contributing to dispersion interactions than in the underlying CNT molecule. Furthermore, many of these atoms are hydrogen atoms, which have a considerably smaller dipole polarizability than carbon atoms [62]. This explains the 20% difference between the stabilization energies and the fact that solid CNT cannot be dissolved by simple aromatic solvents. Note that solid CNT produced in electric arcs is amorphous, it consists of randomly arranged nanotubes or bundles of nanotubes. In our studies, we did not consider such irregularly arranged nanotubes, instead we studied only clusters of crystalline CNTs.

**Figure 6:**
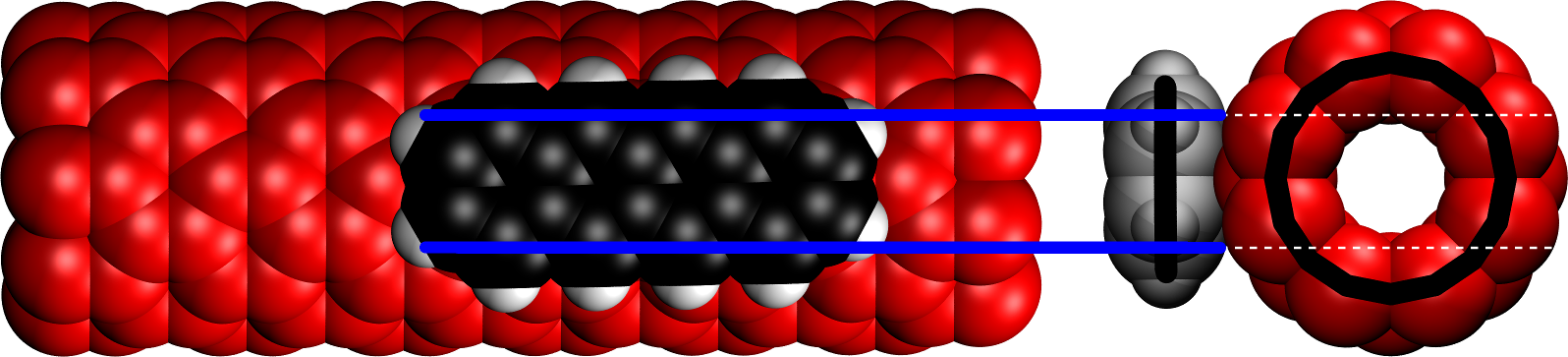
The contact zone of an (8.0)-CNT/tetracene complex. The bold black lines in the traverse section represent the positions of the nuclei. Reprinted with permission from [44], copyright 2017 Elsevier.

In a second paper [14], we studied the dependence of the stabilization energy on the number of atoms for a series of six aromatic and polyaromatic molecules benzene, naphthalene, anthracene, phenanthrene, pyrene and tetracene with a (8,0)-CNT molecule, see Figure 6 for the (8.0)-CNT/tetracene complex. For the series of acenes with the growth direction parallel to the CNT molecular axis, we found an excellent correlation with the number of carbon atoms. The energies for phenanthrene and especially for pyrene were, however, not well reproduced by the regression function because the shape of these molecules, and therefore the area of the CZ, is different from that of the four acenes. We also showed that when a planar molecule comes into contact with a CNT, it will bend towards the CNT, and this increases the number of atom pairs in close contact or, in other words, the size of the CZ.

### Adhesive forces

Although CNTs cannot be dissolved in common organic solvents, it is possible to achieve dissolution by adding small amounts of a third substance, i.e., a solubilizer. Very different substance classes are claimed to be efficient solubilizers and the main question is: How can small amounts of these substances achieve separation of CNT molecules from the bulk solid? A comparison of the total energies of the systems with and without solvated CNT molecules does not explain the process of separating CNT molecules from the bulk. This can only be done with the help of forces. Every CNT molecule in the bulk is a subsystem in a large interacting system that is stabilized by adhesive forces. A CNT can be separated from the bulk only if the pull-off force is larger than the maximum adhesive force. A satisfactory explanation of the process of dissolution must include not only the origin of such pull-off forces but also show which point an external force can act on. For answering both questions, papers from the group of Nakashima [63–64] provide valuable insights. The solubilizers used by these researchers to dissolve bulk CNTs consisted of an aromatic moiety with at least three condensed aromatic rings connected by a very short aliphatic chain to a so-called solvophilic group, which could have a very different polarity. Embedding the solvophilic group into the solvent bulk is essential for the solubilizer to facilitate dissolution. Nonpolar solvophilic groups enabled CNT molecules to be dissolved in nonpolar solvents, while strongly polar or charged solvophilic groups allowed the CNTs to be dissolvated even in polar solvents. The aromatic moiety, on the other hand, is attached to a CNT molecule. The collisions of solvent molecules and the solvophilic moiety result in the generation of stochastic impulses that may add up to a net pull-off force that acts via the solvophilic group and the short connecting chain on the aromatic moiety. If the maximum adhesive force in the solubilizer/CNT(molecule) system is larger than the pull-off force, the solubilizer will not be separated, but the pull-off force will act on the CNT molecule and try to pull it off the bulk. This will happen if the maximum adhesive force in the CNT(molecule)/CNT(bulk) system is smaller than the pull-off force. Then the solubilizer is efficient. One can assume that more than one solubilizer molecule will stick to a CNT molecule and that external forces acting via several solubilizer molecules will separate a CNT molecule from the bulk. After separation of the CNT from the bulk, the solubilizer molecules will remain attached to the dissolved CNT molecule and thus avoid immediate aggregation.

**Figure 7:**
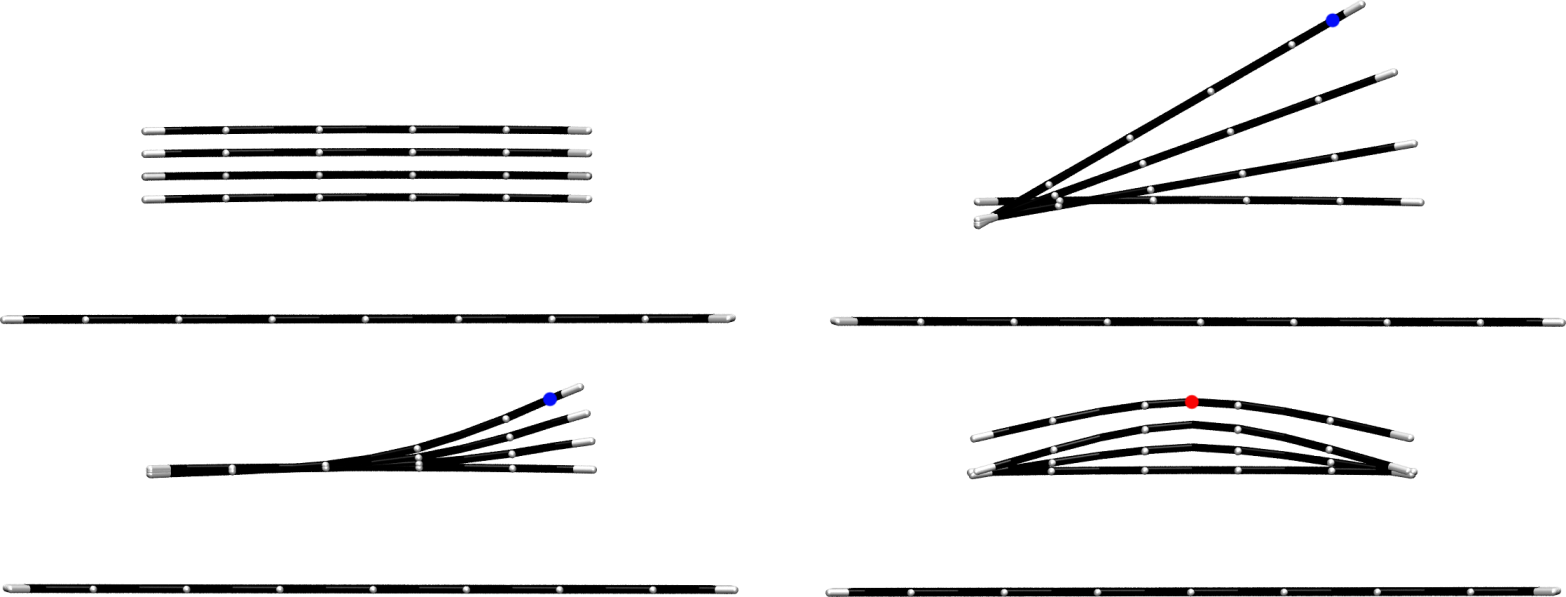
The separation of tetracene from graphene. Top row: Mode S1 (left), mode S2 (right). Bottom row: mode S3 (left), mode S4 (right). The blue and red dots indicate the pull-off points.

**Figure 8:**
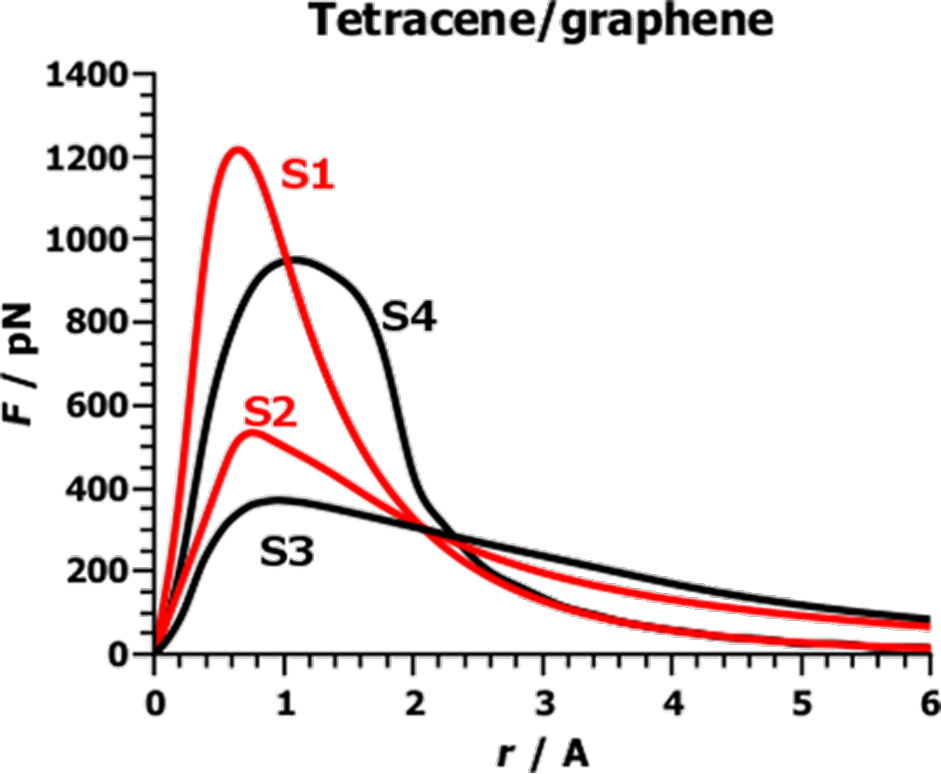
The slope functions for the separation of tetracene from graphene for the four separation modes. Red: Rigid adsorbate. Black: Flexible adsorbate.

To find out how the efficiency of a Nakashima-type solubilizer depends on the number of condensed aromatic rings in the aromatic moiety, we calculated the adhesive force functions for the separation of benzene, anthracene, tetracene and pyrene adsorbed to (8,0)-CNT and graphene [14–15,44]. The basic features are best understood by considering the separation of an adsorbate from graphene, where, in the equilibrium geometry, the CZ is the intersection of the area of the adsorbate and the graphene sheet, and is, therefore, proportional to the area of the adsorbate. An adsorbate can be rigid or flexible, and the pull-off point can be at the edge or in the middle of an adsorbate. Thus, four different separation modes can be formulated. Figure 7 shows the four separation modes for the separation of tetracene from graphene, Figure 8 shows the slope functions for the four separation modes.

1. In separation mode S1, a rigid adsorbate is separated so that the distances of all atom pairs in the CZ increase by the same factor *f*. Think of the separation of two glass plates in contact without tilting. In such a separation, the interaction across the whole CZ changes equally, and, therefore, the reduced CZ is identical to the CZ, which is zero as soon as the separation is larger than the threshold value. Since all atom pairs contribute equally to the change in the interaction energy, the adhesive forces are directly proportional to the size of the CZ.

2. Separation mode S2 can be regarded as the separation of two glass plates by tilting. The pull-off force acts at one end of the rigid adsorbate and causes a rotation about the pivot at the other end. The distances of all atom pairs in the CZ increase at different rates. For each fixed tilt angle, the factor *f* is proportional to the tilt angle and the distance of the moving atom from the pivot. The distances of all atom pairs increase linearly along the length of the adsorbate, while the atom pairs furthest from the pivot reach the threshold value first, after which these atom pairs no longer contribute to the adhesion energy. The reduced CZ is maximal. Only the distances between the pivot atom pairs remain constant. In this separation mode, the position of the adsorbate changes from initially parallel to orthogonal with respect to the adsorbent. Only then is the adsorbate separated from the adsorbent. The adhesion energy changes less strongly than in mode S1 and, accordingly, the adhesive forces are smaller. Note that, in molecular systems, the pivot is slightly shifted.

3. Separation mode S3 is similar to separation mode S2, in that the external force acts at the edge of the adsorbate. However, in S3 the adsorbate is flexible, and bends during separation (peeling). Therefore, in all atom pairs far from the pull-off point, the distances remain largely unaffected. These atom pairs form the CZ and contribute most to the adhesion energy, but not at all to the adhesive forces. The pair distances of all other atom pairs are stretched; if the stretched distance is shorter than the threshold value, the adhesive forces resist the pulling, and in this region the adsorbate is bent. The atom pairs of the convex adsorbate form the reduced CZ. For distances larger than the threshold, no adhesive forces resist the pulling, and the adsorbate relaxes. See Figure 5. During relaxation, the bending energy is released. By continuously pulling at the pull-off point, the non-interacting part of the adsorbate increases steadily. The CZ is steadily reduced but remains as large as possible, and the small bent area of the adsorbate, i.e., the reduced CZ, remains approximately constant in size and moves towards the pivot. The change in the stabilization energy of the reduced CZ is not only due to the separation of the atom pairs but also due to the bending of the adsorbate. The energy needed for doing this, the bending energy, is stored in the adsorbate. When the pair distance is larger than the threshold value, the bending energy is released during relaxation of the adsorbate. The change in the stabilization energy therefore depends strongly on the stiffness of the adsorbate. A flexible adsorbate can be easily bent. The bending energy stored in the adsorbate is small and therefore only little bending energy will be released. The reduced CZ is small. For a stiff adsorbate, the bending energy and the reduced CZ are large. For infinite stiffness of the adsorbate separation mode S3 becomes separation mode S2.

4. In separation mode S4, the external force acts at the middle of the flexible adsorbate. Only the atoms close to the non-terminal pull-off point are displaced. The reduced CZ is symmetrical to the pull-off point, and the CZ is farther away. If only the distances of the atom pairs close to the pull-off point increase, then sufficiently large adsorbates are bell-shaped, which means that the center of the adsorbate is concave, further out, it is convex. This causes strong bending of the adsorbate and a substantial reduction in the stabilization energy. Although only small parts of the CZ are reduced, the increase in the bending energy makes this separation mode less favorable than S3 but still more favorable than S1. In separation modes S1, S2 and S3, dragging and thus friction can be avoided. In mode S4, the left and right wings will always slide over the adsorbent unless stretching of the adsorbate is less costly than dragging the parts into contact with the adsorbent. For infinite stiffness of the adsorbate, separation mode S4 becomes separation mode S1.

The slope functions for the separation of tetracene from graphene are shown in Figure 8. Small and isotropic adsorbates such as benzene or pyrene are stiffer than long, anisotropic acenes and bending costs more energy. Only in complexes with large adsorbates will a large part of the complex be nearly parallel to the adsorbent, causing bending (mode S3) instead of tilting (mode S2). For mode S1, we found an increase in the maximum adhesive force with the number of aromatic rings. The force increases from 451 pN (benzene) through 962 pN (anthracene) and 1059 pN (pyrene) to 1219 pN (tetracene), For mode S3, the force increases from 214 pN through 353 pN (anthracene) and 362 pN (pyrene) to 371 pN (tetracene). This demonstrates, firstly, that in mode S1, the reduced CZ increases with the size of the adsorbate. Therefore, the maximum adhesive force is proportional to the size of the adsorbate, and, secondly, that in mode S3, the maximum adhesive force increases strongly from benzene to anthracene, but that the difference between the large adsorbates anthracene, pyrene and tetracene is much smaller than between benzene and anthracene. These observations are in accord with the finding that the aromatic moiety of a Nakashima-type solubilizer should have at least three condensed aromatic rings to be efficient. This connection of size and shape of the adsorbate and its elastic properties is true for all classes of molecules when an adsorbate comes into contact with nonplanar adsorbents, because bending can increase the CZ, improving the stabilization of the complex. If bending produces more stabilization (due to the larger CZ) than it costs, the adsorbate will change its form to maximize both adhesion energy and adhesive forces.

### Hydrophobic interaction, hydrogen bonding and properties of liquids

Condensed-matter properties are strongly influenced by cooperative effects caused by more than two interacting particles (many-body effects). In statistical physics, these effects are represented by the cluster expansions of the partition function or the thermodynamic potentials [65]. The cluster expansion of the interaction potential of a condensed matter system composed of molecules,

[11]
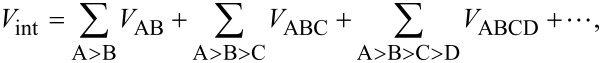


says that the properties of a liquid cannot be described solely by two-body contributions, that is, contributions of two solvent molecules. Clusters of molecules with low spatial symmetry have, in general, several stable structures that vary in their stabilities and molecular properties, e.g., electric multipole moments and polarizabilities, and thus contribute differently to the stabilization energy. Electrostatic interactions are strictly additive for all distances at which the molecular electron distributions do not overlap, all other basis interactions are non-additive and contributions of larger clusters are essential. Whereas long-range dispersion interactions are approximately additive, induction interactions are strictly non-additive. This means that it is not possible to add up all electric fields due to the static moments of the surrounding molecules and then calculate the induction energy for a given molecule. However, in the case of less polar or less polarizable molecules, approximate additivity seems to be reasonable [2]. Liquid alkanes are such systems. All straight-chain alkanes (*n*-alkanes) can be derived from the parent substance methane by substituting one hydrogen atom for *n*-alkyl chains of increasing length. Under standard conditions, macroscopic amounts of alkanes occur in all three phases. *n*-Alkanes with up to four carbon atoms are gases, *n*-alkanes from five to 17 carbon atoms are liquids, and all longer *n*-alkanes are solids. The melting point and particularly the boiling point (BP) reflect the degree of interaction between the molecules in the condensed phase, the degree of interactions itself depends on the size of the CZs. The boiling point is especially significant because it is related to the process of separating molecules from the bulk, i.e., to working against adhesion forces. The melting point is related to the change of the short-range order in the two condensed phases. The dynamic (shear) viscosity is another property that strongly depends on intermolecular interactions. It is related to the resistance of the molecules to moving relative to each other, which is nothing but friction caused by either attractive interactions between the molecules or mechanical locking caused by surface roughness.

All experimental data mentioned in this section were taken from the online databases GESTIS [66], EngineeringToolBox [67] and ChemicalBook [68]. The BP of *n*-alkanes is a monotonically increasing function of the chain length. The changes in BP for the first four *n*-alkanes are rather large: 73 °C between methane and ethane, 47 °C between ethane and propane and 41 °C between propane and butane. The low BP of methane reflects very weak molecular interactions. The largest contribution to electrostatics are octopole–octopole interactions. In the liquid phase, this interaction varies as 1/*r*^7^ with the intermolecular distance. The lowest contribution to induction is the interaction of a static octopole and an induced dipole with a 1/*r*^8^ distance dependence, so the dipole–dipole dispersion interaction with the 1/*r*^6^ distance dependence yields the largest attractive contribution. Due to the free rotation in the gas phase, the electrostatic interaction is of much shorter range, the thermal averaged interaction is proportional to 1/*r*^14^. In ethane, the lowest multipole is a quadrupole with a very low quadrupole moment. Accordingly, contributions to electrostatics and induction are also very small, and dispersion again yields the largest contribution. But in this case, the size of the CZ becomes important. The more atoms with large polarizability there are in the CZ of an atom, the larger is the dispersion contribution, and the polarizability of the carbon atom is much larger than that of the hydrogen atom. In a large alkane molecule, many more carbon atoms are close to each other because of the short covalent bonds between them, in contrast to the large distances between the carbon atoms in liquid methane. In the latter case, any atom seeing a carbon atom will see also some hydrogen atoms but no other carbon atom close by, whereas an atom seeing a carbon atom in a large alkane molecule will probably see a second or a third carbon atom. The CZ of an atom seeing methane molecules is much smaller than that of an atom seeing large alkanes. Therefore, a larger number of carbon atoms in the CZ will increase the stabilization much more than the same number of hydrogen atoms. The differences in the boiling points of the first few *n*-alkanes also show a strong influence of the shape of the molecules on the CZs. Methane is much less anisotropic than ethane, which itself is less anisotropic than propane. However, the degree of anisotropy becomes less important, the larger the alkane chain is. Then, the size of the CZ becomes decisive, and the successive increases in BP become roughly constant. The importance of the anisotropy of the interacting molecules and, consequently, of the shape of the CZ, can be seen in the differences in the boiling points of isomers of a certain alkane. Straight-chain isomers can lie parallel to each other, achieving a larger CZ than branched, globular molecules. The boiling points of *n*-pentane, isopentane and neopentane are 40 °C, 28 °C, and 10 °C. On the other hand, all disk-shaped, cyclic alkanes have higher boiling points than the straight-chain molecules. The boiling point of cyclopentane is 49 °C. Likewise, the boiling points of *n*-hexane and cyclohexane are 69 °C and 81 °C, respectively.

The dependence of friction on the size of the CZ also explains why the viscosity of straight-chain alkanes increases with chain length. Surface roughness and mechanical locking are a second cause of viscosity, and they explain why the viscosity of large branched alkanes is larger than that of the corresponding straight-chain alkanes. The much larger CZ of disk-shaped cyclic alkanes such as cyclopentane, cyclohexane or cycloheptane explains the larger dynamic viscosity of cycloalkanes compared to that of straight-chain alkanes. The importance of both causes is nicely demonstrated by the viscosities of cyclohexane (1.20 mPa·s), benzene (0.65 mPa·s) and *n*-hexane (0.33 mPa·s). The cyclohexane molecule is disk-shaped and, because of the axial CH groups, has a higher roughness than the benzene molecule. Linear *n*-hexane, finally, has the smallest CZ of the three molecules. The shape and size of the CZ also explains the low viscosity of spherical molecules, such as neopentane, or quasi-spherical molecules, such as isopentane and isohexane, compared to *n*-pentane, *n*-hexane or *n*-heptane [69–71], which is surprising when one assumes that branched alkanes always have a higher viscosity. MD results for the pentane isomers are 0.2667, 0.2445 and 0.1500 mPa·s for *n*-pentane, isopentane and neopentane, respectively [70]. Experimentally, the viscosity of branched isobutane (0.166 mPa·s) was found to be slightly larger than that of *n*-butane (0.162 mPa·s) [71]. However, one can speculate that this small difference in the viscosities of the two isomers is not caused by branching but by the disk-shape of isobutane.

Since the largest contribution to the interaction between alkane molecules is the dispersion interaction, it is not only responsible for liquefaction and solidification of alkanes but also for the stabilization of hairpin structures of large *n*-alkanes with about 18 to 20 carbon atoms. In this conformation, the CZ, and hence the attractive interaction, is maximized for the carbon atoms in the arms. Only the atoms in the loop of the hairpin are further away from other atoms and, moreover, the carbon skeleton in the loop is strongly bent, which destabilizes the hairpin structure. Only if the arms are long enough and the interaction between them outweighs the destabilization in the loop does the hairpin become the most stable structure. One can assume that the stabilization of these conformations is only important in the gas phase, because in a liquid, every alkane molecule will be in contact with several other alkane molecules, which makes the interaction between non-bent alkane molecules more probable. This dispersion-dominated interaction between alkane molecules is the physical origin of the so-called hydrophobic interaction.

**Figure 9:**
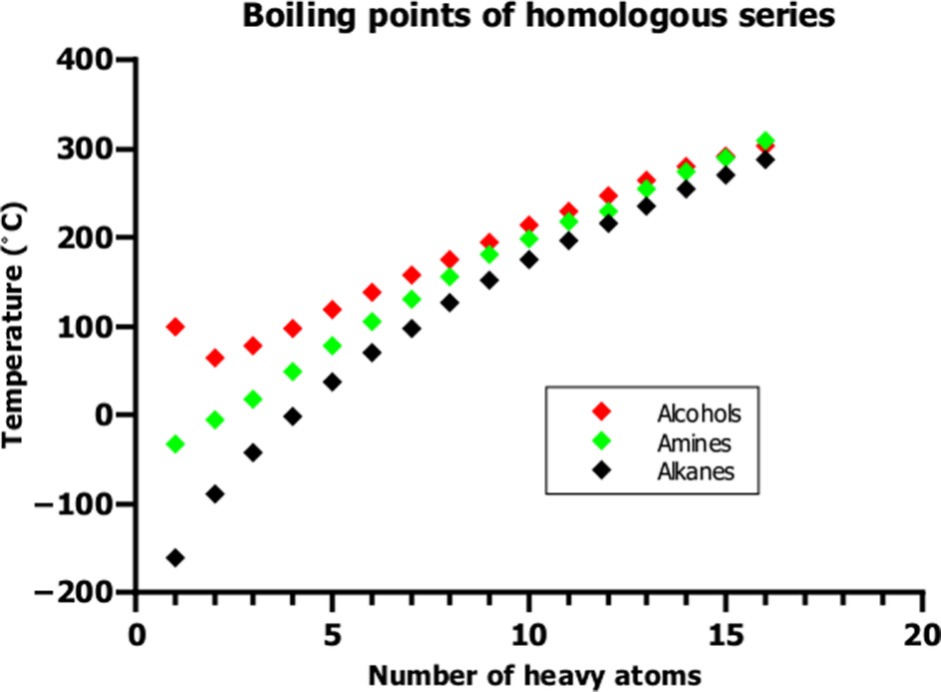
Boiling points of straight-chain primary alcohols, straight-chain primary amines and straight-chain alkanes. Heavy atoms are C, N or O.

Polar liquids are systems in which many-body contributions cannot be neglected because of the non-additivity of polarization effects especially for induction interaction. We discuss straight-chain primary alcohols and straight-chain primary amines and their corresponding parent substances, water and ammonia. Figure 9 shows 1) that primary alcohols exhibit a higher BP than primary amines and alkanes having the same number of heavy atoms (C, N or O); 2) an apparent convergence of the BPs of amines, alcohols and alkanes with increasing size of the molecules; 3) large differences between the BPs for the respective small members of the homologous series; and 4) an exceptional BP of water, the parent substance of the alcohols. Except for water, the BPs of all three series increase monotonically with increasing size of the molecules. Ammonia and the first two amines are gases, amines with three to twelve heavy atoms are liquids, while all higher amines are solids at room temperature. Water and all alcohols with up to twelve heavy atoms are liquids, all higher alcohols are solids at room temperature. The difference in the BPs of the parent substances methane and ammonia is about 130 °C; between ammonia and water, the difference is a further 130 °C. Due to the different shapes and volumes of the three parent molecules, the densities of the liquids are rather different: H_2_O (1.00 g/cm^3^), NH_3_ (0.73 g/cm^3^) and CH_4_ (0.42 g/cm^3^). This implies for the number of molecules in a certain volume a ratio of 1:0.77:0.47. Accordingly, the average intermolecular distances in ammonia is 9% greater than in water, but 28% smaller than in methane. In other words, the attraction between ammonia molecules is much smaller than that between water molecules, but greater than the attraction between methane molecules. The water molecule has a larger dipole moment (1.85 D) than ammonia (1.47 D) and the anisotropies of the quadrupole moments are very different. For water the quadrupole components are (−2.12, 2.32, −0.20) DÅ, whereas for ammonia they are (1.27, 1.27, −2.54) DÅ. The shape of the water molecule, the magnitude of the multipole moments and the anisotropy of the quadrupole moment together with the shorter distance between the molecules enable much stronger intermolecular attractions in liquid water than in liquid ammonia. This is also in line with the fact that the magnitude of the dispersion contribution, *E*_D_, in the equilibrium structures of the dimers of water, ammonia and methane varies as follows: *E*_D_(water) *> E*_D_(ammonia) *> E*_D_(methane) [8,72], although the magnitude of the dipole–dipole polarizabilities shows the inverse trend: α(H_2_O) = 1.501 Å^3^ *<* α(NH_3_) = 2.103 Å^3^ *<* α(CH_4_) = 2.448 Å^3^. This means that at equilibrium short-range dipole–quadrupole and quadrupole–quadrupole dispersion contributions are more important than the long-range dipole–dipole dispersion contributions. Substitution of one hydrogen atom by a methyl group in each parent molecule increases the size of the molecule and thus also the long-range dispersion interactions. This causes the larger BP of ethane, but it does not outweigh the loss of attractive interactions in methanol relative to the interaction in water, and thus causes the lower BP of methanol. In the amine series, we see that the increase in the molecular size is more important than a possible reduction of electrostatic, induction and short-range dispersion interactions. Further increasing alkyl chains leads to increasing BPs in all three homologous series.

In ice and, to a lesser extent, also in liquid water, each water molecule is surrounded by four other water molecules such that their dipole moments do not cancel each other out. Due to the resulting dipole-induced dipole interaction, many-body contributions, especially three-body contributions, dominated by the induction energy, are essential [2]. In methanol, the alkyl groups disturb the short-range order found in water. The mean distance between the OH groups is increased and, accordingly, the attractive electrostatic and induction interactions are reduced. Although the volume of the methanol molecule is larger than the volume of the water molecule, and the dispersion interactions are increased, the total interaction is decreased and the BP is lower. With increasing length of the alkyl groups, the BP increases again, *n*-propanol having a BP nearly equal to that of water (97 °C). In chemistry, system stabilization is preferably attributed to local molecular substructures. The most prominent example is the successful explanation of covalent bonding using groups of two atoms. Adopting this localized approach, the stability of water or alcohol dimers can be attributed to a group of three atoms forming a hydrogen bridge, A–H···B, where A and B are atoms with higher electronegativity than the bridging hydrogen atom, and B has an electron lone pair. The claims that charge transfer and covalent bonding are relevant for hydrogen bonding have their origins in this interpretation of bonding with the three-atom-four-electron group using the Lewis structures A–H|B, A|^−^ H–B^+^, and A|^−^ H^+^|B. However, these Lewis structures are simply necessary to describe the polarization of the atom group A^δ−^–H^δ+^···B^δ−^. The latter formulation also facilitates the interpretation that hydrogen bonding is predominantly electrostatic in character. In the MO description, the three Lewis structures are contained in a four-electron-three-MO CASSCF wave function, which is the lowest-level wave function including polarization effects in this atom group. However, all these simple wave functions ignore 1) all atoms attached to atoms A and B, and, in consequence, 2) the electric multipoles of the whole molecules containing A and B, 3) the polarization of the electron density of these molecules, and 4) any kind of dynamic electron correlation that covers dispersion interaction. The quantum chemical models developed to explain strong chemical bonding in localized regions of a molecule cannot cover the non-localized and non-additive bonding contributions that are typical for WMI. Using them to explain WMI leads to serious errors.

In two papers [7–8], we investigated the contributions of the four basic interactions to the stabilization of alcohol and amine dimers, demonstrating that the whole molecules contribute to the stabilization, not just the atoms of the central moiety A–H···B. We found that, for the hairpin structures of amine dimers R^1^-NH–H···NH_2_–R^2^ with up to four carbon atoms, the stabilization energy increases by a constant increment for each added CH_2_ group. For the stretched hairpin structures going from the ammonia dimer to the methylamine dimer gave a substantial stabilization, but any further growth of the alkyl chains did not improve stabilization. In the alcohol series, R^1^-O–H···OH-R^2^, we investigated only the stretched hairpin structures and found improvements of the stabilization up to the ethanol dimer, but no change for the higher alcohols. These findings agree with those for the CZ of alkanes. In the hairpin structure each carbon atom in one arm is close to the corresponding carbon atom in the opposite arm and, thus, the CZ is maximal, and increases with each inserted methylene group. In the stretched hairpin structure, only the α and maybe the β carbon atoms, and the attached hydrogen atoms, of one monomer are in contact with the nearest atoms in the opposite monomer. Hence, the CZ is minimal. This is true for the growth of straight-chain alkyl substituents. However, in *tert*-butylamine dimer or the *tert*-butanol dimer, each primary methyl group is equally close to the oxygen or nitrogen atom of the opposite monomer, but the distances between the methyl groups of the two monomers are larger than the distances between opposite carbon atoms in the hairpin structure. Accordingly, we find the following order of dimer stabilities: *n*-butyl dimer(hairpin) *> tert*-butyl dimer *> n*-butyl dimer (stretched hairpin). The higher stability of the hairpin structures due to intramolecular interaction is, however, not relevant for the properties of the liquids, which depend on intermolecular interactions, and we do not even know whether two-body clusters in the cluster expansion are indeed dimers. But we do know for sure that the BP of *tert*-butanol (83 °C) is 35 °C lower than the BP of *n*-butanol and that the BP of *tert*-butylamine (45 °C) is 33 °C lower than the BP of *n*-butylamine. This is comparable to the 30 °C difference between the BPs of *n*-pentane and neopentane.

Comparison of the viscosities of alkanes with those of alcohols and amines is difficult because far less experimental data are available for the latter two groups of substances, and the data found in the literature vary considerably. Nonetheless, the available data do allow the following conclusions to be drawn: First, polar groups increase the viscosity due to electrostatic and induction interactions, as the values for *n*-pentane, *n*-butylamine and *n*-butanol show, which are 0.240 mPa·s (25 °C), 0.470 mPa·s (20 °C), and 2.95 mPa·s (20 °C), respectively. The assumption that the increase in viscosity is caused by hydrogen bonding is unjustified. Replacing a CH_2_ group in cyclopentane by a sulfur atom doubles the viscosity from 0.413 mPa·s (25 °C) to 0.973 mPa·s (25 °C) [73]. Second, the combination of polar groups and branching enhances the increase in viscosity. The viscosity of isobutanol (3.95 mPa·s at 20 °C) is considerably larger than the viscosity of *n*-butanol. These data show that any attempt to attribute properties of condensed-phase systems to a single cause, e.g., hydrogen bonding, fails.

### Bonding in cellulose

Cellulose is a material showing polymorphism, crystals of the Iα and the Iβ allomorphs are composed of layers of parallel aligned cellulose chains, which are chains of D-glucose rings connected by 1→4 glycosidic bonds. In each glucose ring, there are five axial CH groups, and two OH groups and one hydroxymethyl group in equatorial position. The cellulose chains are stabilized by intrachain hydrogen bridges, while interchain hydrogen bridges connect the cellulose chains. All equatorial OH groups are involved in intra- and interchain hydrogen bridges. In addition to these hydrogen bridges, the hydroxymethyl group can also contribute to intersheet hydrogen bridges. This flexibility is due to the different possible conformations the hydroxymethyl group can adopt. It is common belief among cellulose scientists that this hydrogen-bonding network is responsible for the stability of cellulose [74–75] and also for the insolubility of cellulose fibers, but this view has recently been criticized [76]. After all, the crystal structures of cellulose Iα, cellulose Iβ and cellulose II vary considerably and so do their hydrogen bonding networks. Only in cellulose II can one speak of a three-dimensional network. In cellulose I, hydrogen bridges are nearly exclusively found within the sheets, with only very few hydrogen bridges connecting the sheets. These so called hydrogen-bonding networks are, however, neither unique nor static [75,77]. MD simulations show that hydrogen bridges are dynamically created and broken [77]. While cellulose Iα is made of one type of layers, cellulose Iβ is composed of two types of sheets, each of which seems to favor a different hydrogen-bonding network [75]. The few intersheet hydrogen bridges cannot explain the stability of cellulose, so other attractive interactions must be responsible for the attraction of the sheets, which in Iβ crystals have Miller indices (100). The (100) surfaces are described as hydrophobic because they are dominated by the axial CH groups (57% accessibility). Both the hydroxyl and acetal oxygen atoms are lying deeper in the sheet and are, consequently, less accessible (43% accessibility) [78]. The interaction between the stacked glucose rings is dominated by dispersion interactions, similar to the interaction between cyclic alkanes, but there are, of course, also electrostatic and induction contributions from all atoms involved, not just from the interaction between CH groups and oxygen atoms. Nonetheless, the importance of dispersion interactions can easily be seen when important properties of all four principal cleavage planes of cellulose [79] are investigated, such as the surface energy, the attachment energy or the surface roughness. In Iβ crystals, these planes have Miller indices (100), (010), (110) and (1−10). The accessibility of oxygen atoms on the surface increases the electrostatic and induction interactions. Consequently, the surface energies of the four surfaces vary by a factor of almost two, according to molecular dynamics studies [78]. The surface energy of the most hydrophobic (100) surface is about 190 mJ/m^2^ whereas for the most hydrophilic (010) surface it is about 350 mJ/m^2^ [78]. Likewise, the energies for the attachment of a new cellulose layer, which is a measure for the cohesive energy, were found to be about 125 kcal/mol for the (100) and about 270 kcal/mol for the (010) surface [78]. These are the energies for the unrelaxed surfaces. Thus, electrostatics and induction only enhance the interaction between different crystallographic planes by a factor of two, which demonstrates the importance of dispersion interactions for the stability of cellulose crystals. This motivated us to make an analogy between bonding in hydrogen-bridged systems and reinforced concrete, which is made of concrete and rebars. The role of concrete can be seen as being played by the largely isotropic dispersion interactions, while the anisotropic electrostatic interactions play the role of rebars. Neglecting dispersion is like forgetting the concrete, while neglecting electrostatics and induction would be forgetting the rebars. French recently criticized inconsistent claims such as “cellulose fibers are insoluble because they are held together by hydrogen bonds”, which he calls a truism. He raised the question, “if the three hydrogen bonds per glucose unit in cellulose Iβ […] explain the insolubility, then why is β-glucose, with five conventional hydrogen bonds per glucose unit […] so soluble?” [76]. He concluded: “other factors such as unconventional C–H···O hydrogen bonding and van der Waals interactions must also be important, and the truism does not bring them into consideration”. In our opinion, the above statement is not a truism, which by definition is frequently true. It is simply wrong because it considers only the rebars and forgets the concrete.

The stability of cellulose crystals is, however, not only due to adhesive forces between layers but also due to dry friction, which describes the processes that hinder relative lateral motions of two solid surfaces moving against each other. The cause for dry friction can be strong adhesion, entanglement of the surfaces due to roughness, or strong interaction between localized parts of the surfaces such as heteroatoms or atom groups. Although the (100) surface in cellulose Iβ crystals is the smoothest of the four principal cleavage planes, the planes do not slide against each other as do the graphene sheets in graphite, because there are adhesive interactions between the sheets as well as friction due to the surface roughness caused by the axial CH groups.

### Adsorption to cellulose

Similar to bonding in the cellulose bulk, hydrogen bonding is regarded as the dominant type of interaction responsible for the adsorption of small molecules with polar groups onto cellulose surfaces. We investigated the adsorption of glucose, cellobiose and cellotetraose onto the hydrophilic (100) surface of Iα cellulose and the hydrophobic (100) surface of cellulose Iβ by using the BP86-D2 density functional and the GLYCAM06 force field [80]. For the adsorption of D-glucose onto the hydrophilic Iα surface, the most stable structure was the one in which the glucose ring was perpendicular to the cellulose surface. At least two hydrogen bridges were found for the structure, depending on the method used. Also on the hydrophobic (100) surface of Iβ, a structure with the glucose ring perpendicular to the cellulose surface was most stable, but with increasing size of the adsorbate the situation changes considerably. In the most stable structures of cellobiose adsorbed to both surfaces, the glucose rings are parallel to the surfaces. Structures with perpendicular glucose rings are markedly less stable, and for the adsorption of cellotetraose, this trend is intensified. Although for the small adsorbates, bonding to the hydrophilic surface is markedly stronger than to the hydrophobic surface, this difference vanishes for large adsorbates. All these findings are consistent with an increasing contribution of dispersion interactions with increasing size of the adsorbate, that is, with increasing size of the CZ. This demonstrates that, as we have frequently emphasized [7–8,80], WMIs cannot be described by a single basic interaction. The contribution of electrostatics to the bonding of complexes with hydrogen bridges is large, but it is not sufficient to explain their stability.

## Discussion

In the Discussion we consider only WMIs between finite molecules. A WMI between the two arms in the hairpin structure of a large alkane is an example of an intramolecular interaction. The interactions between the alkyl groups in alcohol or amine dimers in hairpin structures, or between two parallel alkane molecules, are examples for intermolecular interactions. The physical origin is the same in both cases. The strength of the interaction depends only on the size of the CZ. The near-sightedness of the WMI makes it a local interaction, and the approximate additivity justifies the assumption that the WMI is dominated by pair contributions. If the WMI is dominated by dispersion interactions, it depends only on the distance between the interacting atoms. If the interaction between multipoles of low rank (e.g., dipoles) contributes significantly, the WMI will be anisotropic and will depend strongly on the relative orientation of the multipoles. Adhesion is a process where WMIs stabilize a system consisting of different subsystems. It can be described by using energies or forces, but the descriptions are not equivalent. Forces are vital for describing the perturbations of such systems by external forces, as well as for describing the response of the systems to this perturbation. We showed how a change of the pull-off point can influence the magnitude of the internal force holding the system together. We also showed that, in general, the magnitude of adhesive forces does not depend on the whole CZ. Rather, it depends only on that part of the CZ where the attractive interaction changes but is not yet zero. Thus, the elastic properties of extended molecular systems are directly related to internal forces and WMIs. Although dispersion interactions play a dominant role in WMIs, there are fields of chemistry in which dispersion interaction are systematically ignored as soon as polar atom groups occur, such as those involved in hydrogen bridges, at which point all stabilizing interactions are attributed to the hydrogen bridges. In contrast, we found that, in systems such as cellulose crystals, the electrostatic and induction contributions of hydrogen bridges amplify the stabilizing dispersion interactions, a finding that is confirmed by conjectures and observations of others [76]. The stability of cellulose crystals is, however, not only due to attractions between cellulose chains within and between the layers, but also due to friction. In solids like cellulose, static friction can hinder the lateral movement of the layers against each other. In liquids, kinetic friction is responsible for viscosity, static and kinetic friction are types of dry friction. Friction changes the state of motion of the subsystems involved, it always slows down the speed, the (negative) acceleration is due to a force called a friction force. If the relative lateral motion is due to a pulling or pushing force, in engineering this is called the “load”, the system responds with an opposing friction force. This friction force is directly proportional to the applied load. Friction forces are not conservative and cannot be derived from a potential. Detailed information about friction can be found, e.g., in the Handbook of Tribology [81]. The cause for dry friction can be strong attraction between the surfaces. This strong attraction can be due to adhesive forces, entanglement of the surfaces due to roughness, or strong interaction between localized parts of the surfaces, such as heteroatoms or atom groups. The relation between the magnitude of the friction force, *F*_f_, and the magnitude of the adhesive force *F*_a_ is *F*_f_ ≤ μ*F*_a_. The constant μ is the coefficient of friction and is an empirical quantity of the interacting materials. In most cases it is smaller than 1 [82]. The viscosity of a liquid is caused by friction between the molecules of the liquid. The higher viscosity of branched alkanes compared to linear alkanes can be attributed to entanglement of the molecules, while the higher viscosity of alcohols or amines relative to alkanes is mainly caused by the interactions of the polar atom groups with the other molecules. In any case, all these interactions are WMIs, and in many cases they are dominated by dispersion interactions. Irrespective of how the WMIs in given systems are composed, WMIs are responsible for many properties of condensed matter systems, and forces are important for their description. For the description of processes such as friction or the stability of interfaces, forces are indispensable.
